# Data-driven optimization of the *in silico* design of ionic liquids as interfacial cell culture fluids

**DOI:** 10.1080/14686996.2024.2418287

**Published:** 2024-10-21

**Authors:** Jun Nakanishi, Takeshi Ueki, Sae Dieb, Hidenori Noguchi, Shota Yamamoto, Keitaro Sodeyama

**Affiliations:** a Research Center for Macromolecules and Biomaterials, National Institute for Materials Science (NIMS), Tsukuba, Japan; b Graduate School of Advanced Science and Engineering, Waseda University, Tokyo, Japan; c Graduate School of Advanced Engineering, Tokyo University of Science, Tokyo, Japan; d Graduate School of Life Science, Hokkaido University, Sapporo, Japan; e Center for Basic Research on Materials, NIMS, Tsukuba, Japan; f Research Center for Energy and Environmental Materials (GREEN), NIMS, Tsukuba, Japan

**Keywords:** Word; cell scaffold, ionic liquid, data-driven science, sustainability, mechanotransduction

## Abstract

As an alternative to conventional plastic dishes, the interface between water-immiscible hydrophobic fluids, such as perfluorocarbons and silicones, permits cell adhesion and growth. Thus, it is expected to replace the petroleum-derived products in a sustainable society. However, most hydrophobic fluids are cytotoxic, which limits the range of mechanical and chemical cues exposed to the cells. Using a data-driven approach, this study aimed to identify non-cytotoxic ionic liquids (ILs) as fluid culture platforms to take advantage of their ‘designer’ nature for broadening the possible physicochemical ranges exposed to cells and their repeated use owing to their high heat stability before their biological applications. The new candidates within the readily synthesized ammonium-type ILs were identified through the active cycle of regression and a limited number of cytotoxicity tests. Structure – cytotoxicity analysis indicated that the presence of multiple long alkyl branches was critical for low cytotoxicity. Particularly, we successfully cultured human mesenchymal stem cells (hMSCs) at the trihexylethylammonium trifluoromethylsulfonylimide interface and repeated their use after solvent extraction and heat sterilization. This study identified non-cytotoxic ILs that fulfill plastics’ **3 R** (**R**educe, **R**ecycle, and **R**eplace) requirements and opens new avenues for hMSC fate manipulation through mechanotransduction.

## IMPACT STATEMENT

Materials informatics broadened non-cytotoxic ionic liquids useful for interfacial cell scaffold, which will fulfill plastic’s 3 R requirements and expand the repertoire for mechanobiological manipulation of stem cell fate.

## GRAPHICAL ABSTRACT

**Figure:**
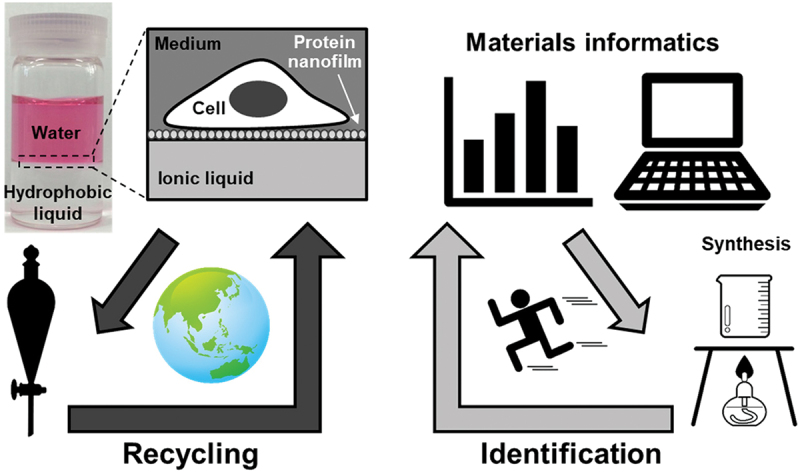

## 1. Introduction

Mammalian cells are conventionally cultured on plastic dishes. These plastic products are disposable to avoid contamination of the cells by bacteria and fungi, which opposes the recent green transformation (GX) trend [1]. Additionally, from a geoenvironmental perspective, marine plastic waste is a critical issue [2]. Therefore, a growing demand exists to **r**educe, **r**ecycle, or **r**eplace (3**R**s) these plastics derived from fossil fuels in the laboratory [3]. Replacing conventional petroleum-based plastics with bio-derived ones (**R**eplacement) is one of the ongoing approaches to address these issues [4]. Culturing cells at the surface of plastic microsphere suspensions to overcome low culturing efficiency in conventional flat surfaces suffering from large volume-to-surface ratio is another approach [5]. This microcarrier culturing can be scaled up into microreactors, eventually reducing the use of plastic products and culture media containing animal-derived nutrients (**R**eduction). These two common ideas stem from the fixed concept that solid supports are indispensable for cell culturing, whose original idea was proposed almost a century ago [6]. Culturing cells at the interface of two immiscible liquids is one of the promising strategies to mitigate this stereotypical view [7,8]. Proteins or phospholipids added to the aqueous culture media self-assemble at the fluid interface using perfluorocarbons (PFCs) as the hydrophobic underlayer and mature into nanofilms, which are robust enough to sustain cellular traction forces [9–12]. Moreover, by suspending the PFCs in culture media, the cells can be grown on PFC droplet surfaces in oil-in-water emulsions, instead of plastic microcarriers, fulfilling two of the three 3 R requirements (**R**eplacement and **R**eduction) [13,14]. PFCs are ideal liquids for such applications because the U.S. Food and Drug Administration (FDA) has approved their inertness toward biological systems [15]. However, fluorinated compounds, such as perfluoroalkyl substances and polyfluorinated substances (PFAS), have extremely low degradability, and their persistence in the environment is becoming a global environmental issue that may affect the food chain, thereby affecting human health and animal and plant habitat and growth [16]. Therefore, their replacement with less environmentally hazardous liquids is rapidly proceeding in the pharmaceutical and semiconductor industries [17].

**Figure 1.**
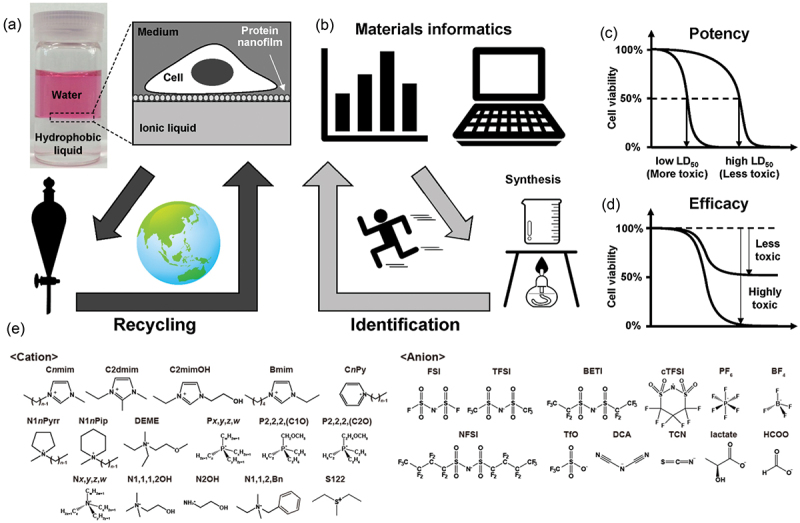
Concept of this study. (a) ‘Greener’ interfacial fluid culture platform. (b) Data-driven identification and designing of non-cytotoxic ionic liquids through active learning. (c,d) Schematic representations of cytotoxicity evaluation in terms of (c) potency and (d) efficacy. (e) Chemical structures of cations and anions focused on in this study.

Recently, we have succeeded in demonstrating that ionic liquids (ILs), room temperature-molten salts purely composed of cations and anions, can be PFC alternatives in the interfacial fluid culture system [18]. Given the low volatility and high decomposition temperature of ILs, they are typically stable at high temperatures of up to 300°C [19]. Hence, they are expected to be recycled after the culture use via dry-heat sterilization. This satisfies the remaining entity of the 3**R**s (**R**ecycling) (Figure 1a). Furthermore, ILs are called ‘designer liquids’ because their physicochemical properties and solvation capability can be tuned to wide ranges depending on the cation and anion species combination [20]. This feature is potentially useful to mechanobiologically manipulate the fate of mesenchymal stem cells (hMSCs) into a specific lineage [11,21] or retain their multipotency [22] for tissue engineering and therapeutic applications without using expensive growth factors and cytokines [23]. Our preliminary screening identified that hMSCs or other mammalian cell lines can be cultured on several alkylphosphonium-type ILs, particularly tributylmethylphosphonium trifluoromethylsulfonylimide (P4441-TFSI) and trihexyltetradecylphosphonium trifluoromethylsulfonylimide (P66614-TFSI). However, we need to identify as many candidates as possible that do not exhibit cytotoxicity to take full advantage of the ‘designer’ nature of the ILs; this is the minimum requirement for their application to cell culture. Particularly, alkylphosphonium-type ILs have been prepared using alkylphosphines as precursors, which are generally volatile and produce foul odors. Moreover, they are highly reactive and easily oxidized and ignited, making them challenging to handle in air. Therefore, exploring candidate substances that are non-cytotoxic, hydrophobic, and easy to synthesize, including precursor safety and handling is necessary to access a cell culture system at the IL interface. However, owing to the infinite number of cation and anion combinations, experimentally assessing cytotoxicity is not realistic.

Herein, we used a data-driven approach to identify non-cytotoxic ILs potentially useful for interfacial cell culture (Figure 1b). This approach can accelerate the investigation via guided experimental design [24] to identify non-cytotoxic ILs with as few experiments as possible. Although *in silico* approaches have been reported to assess the *water-soluble* IL cytotoxicity, most studies have discussed them in terms of potency, such as EC_50_ (half-maximum effective concentration) and LD_50_ (half maximum lethal dose) (Figure 1c), because their potential risk after spilling into biological or environmental aqueous media has been given priority [25,26]. However, such endpoint analysis is unsuitable for our experimental system, where cells are continuously and directly exposed to the *hydrophobic* IL in the biphasic culture system (Figure 1a, right). Therefore, we established a cytotoxicity assessment method suitable for an interfacial fluid culture system, where efficacy becomes highly critical (Figure 1d). We expanded the possible candidates by data-driven optimization using experimentally determined cytotoxicity results of a limited number of ILs as well as the physicochemical and structural features of the ILs (Figure 1e). Our model revealed that ammonium ILs are potential candidates, which was experimentally verified.

## 2. Results and discussion

### 2.1. Cell viability in the IL-saturated culture medium

**Figure 2.**
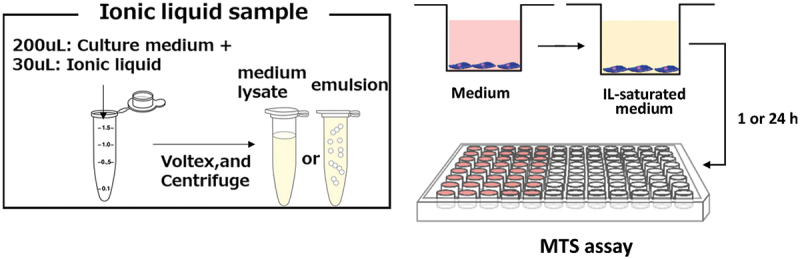
Schematic representation of the procedure for evaluating cell viability against ILs used in this study.

**Figure 3.**
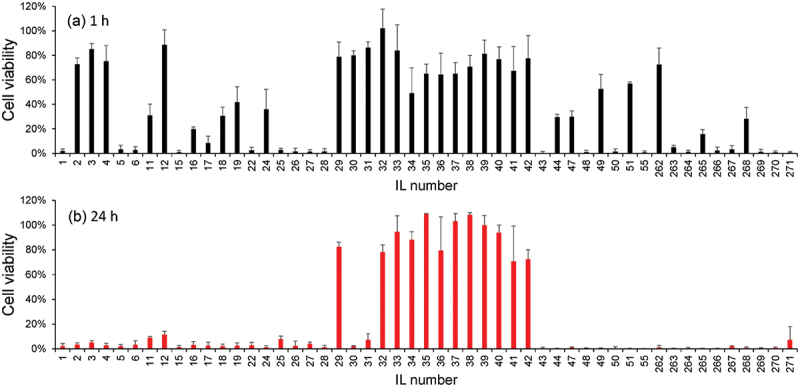
Cell viability analysis based on the MTS assay at (a) 1 h and (b) 24 h incubation. Error bars represent standard deviations of *N* = 3.

Figure 2 and 3 illustrates, respectively, the brief experimental procedure and the results of cell viability experiments at 1 and 24 h after the exposure of hMSCs to a medium saturated with 51 ILs. Despite the cells exhibiting > 60% viability for approximately one-third of the tested ionic liquids at 1 h (Figure 3a), the viability decreased after 24 h incubation (Figure 3b). Therefore, ILs exhibit instant and delayed cytotoxicity depending on their structure. Notably, all the ILs that allowed > 60% viability (12 ILs) were based on the phosphonium-type cations, whereas the ammonium-type cations, which constitute the large portion of the original dataset, were cytotoxic, as we have reported in a previous study [18].

### 2.2. Regression based on cell viability prediction

**Figure 4.**
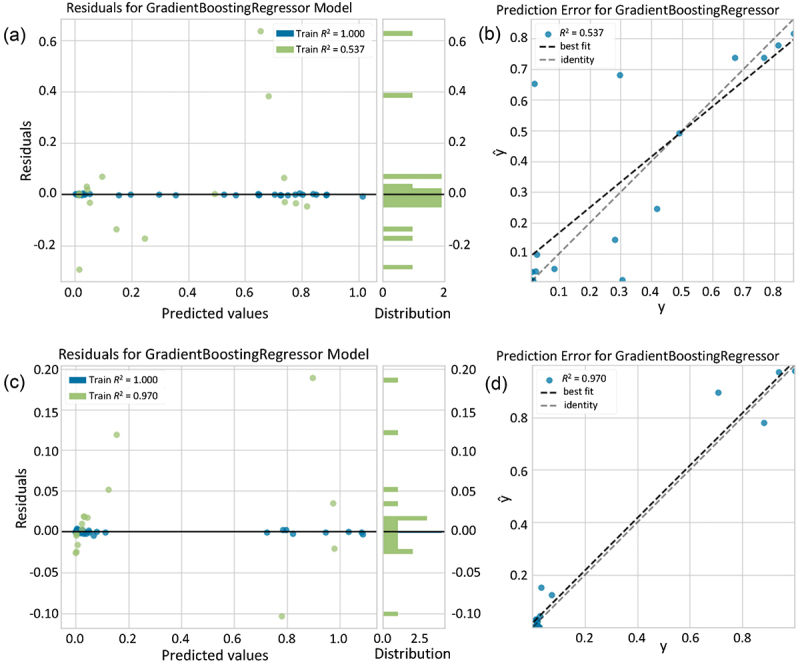
Gradient boosting regressor model evaluation in the case of (a,b) 1 h cytotoxicity and (c,d) 24 h cytotoxicity. (a,c) represents the relationship between predicted value and the residuals (difference between actual and predicted value) in the cases of test and train data. (b,d) show the error in prediction between actual value (y) a predicted value (ŷ) with best fit line.

We compiled two regressors using the method discussed in the method section with a gradient boosting regressor: one for 1 h viability and the other for 24 h viability. In both cases, the regression model was evaluated after separating the data into test and training subsets. Figure 4 illustrates the model evaluation with 5 folds for the 1 h and 24 h cases. The model was evaluated using the test data. Table 1 indicates the evaluation results for the 1 h and 24 h viabilities using several evaluation metrics.

**Table 1.** Regression model evaluation for both 1 h and 24 h cytotoxicity values for ionic liquid. Errors indicate standard deviations during 5 folds training.

	MAE^†^	MSE^†^	RMSE^†^	R^2†^	RMSLE^†^	MAPE^†^
1 h	0.12	0.04	0.21	0.54	0.16	2.76
24 h	0.04	0.01	0.06	0.97	0.04	0.82

^†^MAE: Mean absolute error, MSE: Mean squared error, RMSE: Root mean squared error, R^2^: R-squared, RMSLE: Root mean squared logarithmic error, MAPE: Mean absolute percentage error.

**Figure 5.**
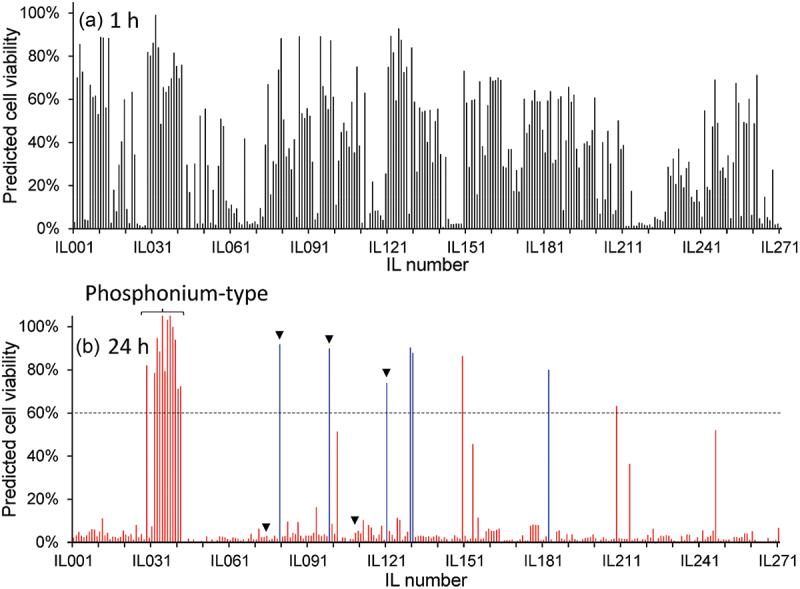
Predicted cell viability for all ionic liquids (ILs) in the initial search space at (a) 1 h and (b) 24 h. The dotted line indicates the viability border (60%) for 24 h prediction. Potentially useful ammonium-type ILs with low cytotoxicity (viability >60%) at 24 h are indicated in blue color. Arrowheads indicate ILs whose cytotoxicity was experimentally verified in Figure 6. ILs indicated by parenthesis in the left side of b are low cytotoxic phosphonium ILs, which are also experimentally verified (Figure 3b).

Figure 5a,b illustrate the viability prediction for 1 h and 24 h, respectively. Surprisingly, when we looked into the data in detail, some of the alkylammonium-type ILs, such as IL80, IL99, IL121, IL130, IL131, and IL183, were suggested to exhibit relatively low cytotoxicity (>60%) even after 24 h (Figure 5b, blue graphs). Compared to the phosphonium-type cations, whose precursors are phosphines, ammonium-type cations, especially tetraalkylammonium cations, can be synthesized from less dangerous molecules in a straightforward S_N_2 reaction. Therefore, more commercial products and derivatives are available that can be easily synthesized. This prediction prompted us to investigate the viability of ILs based on the tetraalkylammonium-type cations. Additionally, our predictions indicated that cholinium-type ammonium-based hydrophobic ILs (IL112, choline-NFSI) are highly toxic, even though choline-type ILs are evidently biocompatible owing to their structural similarity with natural amino acids [27]. However, this result must be further experimentally verified.

### 2.3. Experimental verification of cell viability against the ammonium ILs

**Figure 6.**
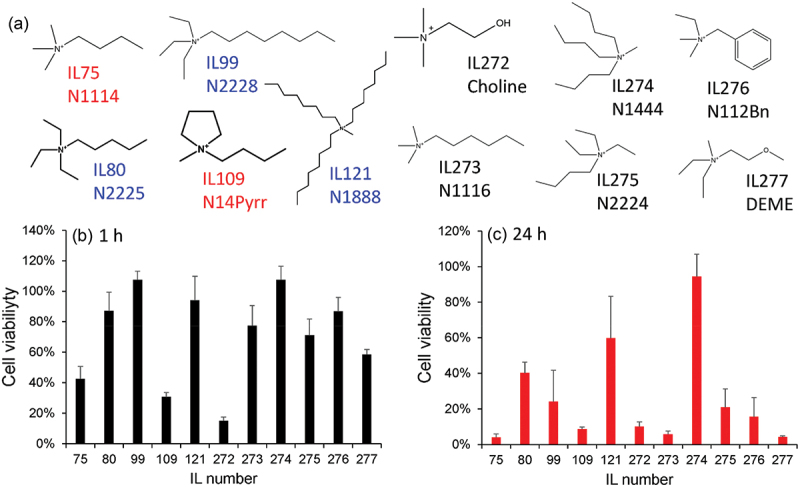
Experimental verification of the cytotoxicity of ammonium-type ionic liquids (ILs). (a) Chemical structures of the cations. The anion was trifluoromethylsulfonylimide (TFSI), except for IL109 (fluorosulfonylimide, FSI). Those shown in red and blue colors are predicted to be toxic (viability <60%) and less toxic (viability >60%), respectively. IL273, 274, 275, 276, and 277 were not listed in the initial dataset; therefore, their cytotoxicity was not predicted. (b,c) experimentally determined viability data of the ammonium ILs at (b) 1 h and (c) 24 h. Error bars represent standard deviations of *N* = 3.

We selected 11 commercially available ammonium-type ILs (Figure 6a) within and outside the initial search space based on the implications of the regression model, and experimentally evaluated their cell viability. The ILs were predicted to be toxic (IL75 and IL109) and non-toxic (IL80, IL99, and IL121) at 24 h in the regression model. Among the tested ammonium-type ILs, only IL121 (N1888) and IL274 (N1444) exhibited moderate-to-low cytotoxicity, even after 24 h of treatment (Figures 6b,c). In another group of tetraalkylammonium-type ILs, including IL80 (N2225-TFSI), IL99 (N2228-TFSI), IL273 (N1116-TFSI), and IL275 (N2224-TFSI), most cells were viable at 1 h but died at 24 h. A careful comparison of the chemical structures of these ILs revealed that the ammonium ILs that exhibited lower cytotoxicity for a longer time (24 h) had multiple long-arm alkyl chains (IL121 and IL273). Contrastingly, the simple elongation of single alkyl chains had a negative effect on cell viability (IL80 > IL99). These results do not agree with conventional understanding, as earlier reviews generally concluded that longer and branched cations exhibit increased cytotoxicity [25,26]. Moreover, ILs containing hydrophilic groups have been shown to be less toxic in earlier reports. However, our results demonstrated poor cell viability with IL containing hydrophilic functionalities, including IL277 (ether) and IL272 (hydroxyl groups). Particularly, the high cytotoxicity of cholinium-type ammonium (IL272) was surprising, even though it was already suggested by our regression model since these ions are widely regarded as low-cytotoxicity cations in aqueous media [28]. These results suggest different structure-cytotoxicity relationships in our experimental results compared to those reported earlier.

### 2.4. In silico *design and cytotoxicity analysis of the ammonium derivatives*

**Figure 7.**
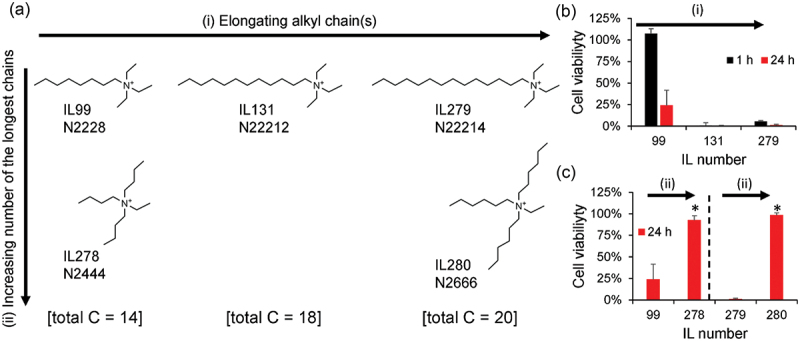
Impact of structural derivatization of the tetraalkylammonium-type ILs on cell viability. (a) Design strategy of the structural derivatization. (i) Starting from IL99 (N2228), the single longest alkyl chain is elongated to IL131 (N22212) and IL279 (N22214). (ii) Starting from IL99 or IL279, the number of the longest alkyl chains is increased from one to three keeping the number of total carbons constant. The anion was fixed to TFSI for all the ILs here. (b,c) Experimental cell viability results for structural derivatization (b) strategy (i) and (c) strategy (ii). Error bars represent standard deviations of *N* = 3. Statistical differences were analyzed using Student’s *t*-test with statistical difference: *p < 0.0001.

The above active learning revealed that some ammonium-based ILs exhibited lower cytotoxicity against hMSCs, and the regression model can predict cell viability to a certain extent. However, a non-negligible gap existed between our experimental data and the conventional understanding, as previously mentioned. Specifically, the number of long alkyl chains and their lengths were important (Figure 6). We created a virtual map (Figure 7a) to directly address this complex structure-cytotoxicity relationship to experimentally address the impact of the alkyl-branched structures of the ammonium cations on cell viability. Starting from N2228-TFSI (IL99), which exhibited delayed cytotoxicity (Figure 7), new ILs were designed in two directions: (i) elongating the longest single alkyl chain or (ii) increasing the number of the longest alkyl chains while keeping the number of carbon atoms constant and keeping the anion unchanged (TFSI). The cell viability became almost zero for the 1 h and 24 h cultures as the length of the single alkyl chain increased from N2228 (IL99) to N22212 (IL131) or N22214 (IL279) (Figure 7b). These results agree with those of an earlier report [29] and our expectations based on the cytotoxicity screening shown in Figure 6. However, when we increased the number of longer alkyl chains from N2228 (IL99) to N2444 (IL278) or N22214 (IL279) to N2666 (IL280), the cytotoxicity decreased (viability increased) significantly for both ILs up to levels almost comparable to that of the control (culture medium only, Figure 7c). These results demonstrate that multibranched tetraalkylammonium with longer alkyl chains (at least butyl) is critical for making ammonium-type ILs less cytotoxic.

### 2.5. *Cell culturing application on pristine or recycled ILs* in silico *design*

**Figure 8.**
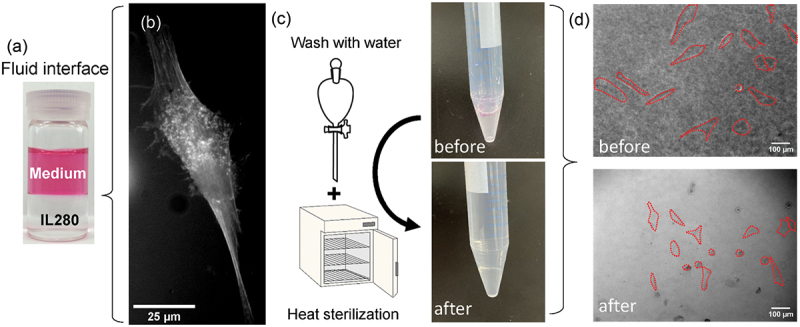
Application to an interfacial fluid culture platform for human mesenchymal stem cells (hMSCs). (a) The fluid interface based on IL280 (N2666-TFSI) were used for culturing hMSCs. (b) A representative image of a hMSC expressing lifeact-GFP, attached at the IL280 interface. (c) Schematic drawings of the recycling procedure and the photographs of the recycled ionic liquid (IL) layers before and after recycling. (d) Bright-field images the hMSCs attached to the IL280 interface (N2666-TFSI) before and after recycling. Cellular outlines were drawn in red to highlight the cellular morphology.

Encouraged by the satisfactory cell viability against multi-branched ammonium-type ILs (IL279 and IL280) comparable to that of the culture medium, we finally attempted to use one of them for interfacial fluid culturing (Figure 8a). Figure 8b illustrates successful cell attachment and growth of hMSCs transiently expressing lifeact-GFP on the N2666-TFSI (IL280) interface. Moreover, when this IL was recycled via solvent extraction against water and heat-sterilized at 100°C (Figures 8c), it could be used again as a culture scaffold in a similar fashion to that before recycling (Figure 8d). To further check the repetition of recycling, IL280 was exposed to the culture medium in the cell culture conditions overnight, followed by washing with water and heat-sterilization. Again, the hMSCs attached to the IL280 interface in a similar fashion to that at the pristine IL280 (Figure S1). By considering commonly accepted stability of ILs [30,31], the IL can be recycled more than three times three times confirmed experimentally here. Therefore, our data-driven optimization successfully identified useful ILs as interfacial cell culture fluids that could potentially fulfil the 3Rs.

### 2.6. Consideration of the structure – cytotoxicity relationship

Current experimental and *in silico* analyses of IL cytotoxicity have shown different structure – toxicity (or safety) relationships from the common trends reported in earlier studies in the following aspects:

1. Hydrophilic functionalities have a negative impact on cell viability.
2. Multiple long alkyl branches are less toxic; however, the mere elongation of a single alkyl chain is not good.

We believe that these differences are essentially attributable to the different methods of toxicity testing used in this study. Considering the interfacial fluid culture system, where cells are continuously exposed to pure IL layers as culture scaffolds, we evaluated the toxicity against the culture medium at the saturated concentration of each IL. Thus, the hydrophilic ILs were dissolved in media far beyond the EC_50_ and IC_50_. In other words, our approach evaluates cytotoxicity in terms of efficacy rather than potency (Figure 1c,d). Therefore, less cytotoxic ILs are mostly composed of hydrophobic anions because hydrophobicity/hydrophilicity is mostly determined by ILs’ anionic structures. This is a plausible reason for the negative effects of the hydrophilic functionalities on cell viability (point (i)). Therefore, cholinium-type ammonium ILs such as IL112 and IL272 exhibit severe cytotoxicity, even though the cations have been shown to be biocompatible in conventional studies [27].

Previous studies have discussed the possible toxicological mechanisms of ILs. One of the most widely accepted ideas is that the lipophilic or high water-from-membrane partitioning nature is critical for ILs’ toxicology induction [32]. Other studies discussed ILs’ interaction with the mitochondrial membranes, resulting in reactive oxygen species production and apoptosis induction [33]. These characteristics are strongly related to the structure of cations, especially on the alkyl chain length and its head group through electrostatic interactions of cation and negatively charged cellular membranes [34]. Particularly, longer alkyl chain cations can be inserted into the hydrophobic center of the cellular membrane, thereby becoming more toxic. This result was also confirmed via molecular dynamic simulations [29]. Due to the nature of our cytotoxicity evaluation setup, substantial differences from the earlier work can be reasonably observed due to the different IL partitioning levels in water and cell membranous structures (point (ii)). Another criterion for IL application to interfacial fluid culture platforms is that they should remain as liquids without crystallization. Extensive research shows a clear link between the IL structures and melting points. Excessive alkyls cause ammonium-type ILs to crystallize, while asymmetric structures lower the melting points [35]. Hence, these patterns may partially contribute to the cytotoxicity in our unique setup.

## 3. Conclusions

Herein, we demonstrate data-driven optimization for designing non-cytotoxic hydrophobic ILs in interfacial cell culture fluids. The data-driven approach eliminated biases from our previous research and previous studies on hydrophilic ILs by other researchers. This approach identified tetraalkylammonium-type ILs as promising for cell culture scaffolds. We identified that the existence of multiple long alkyl branches is crucial for low cytotoxicity by further addressing the structure-cytotoxicity relationships of ammonium-type ILs. Although its exact mechanisms require further investigation, this finding will further increase the repertoire of hydrophobic ILs, which will be beneficial for mechanobiologically manipulating stem cell fate depending on the IL species and applying them for tissue engineering and drug screening. Moreover, the recyclability of N2666-TFSI as a fluid cell culture scaffold was confirmed. This system might have the potential for a new sustainable cell culture system that satisfies the 3Rs by applying liquids to the emulsion culture and suspending them in culture media.

## 4. Methods

### 4.1. Dataset building of the ILs

The search space was set as 271 potential IL candidates composed of ammonium, phosphonium, sulfonium cations, and trifluoromethylsulfonylamide, tetrafluoroborate, and hexafluorophosphate anions (Figure 1e and Table S1). Notably, anions mostly determine the hydrophobicity and hydrophilicity of the ILs. Conversely, cations add to the diversity of the physicochemical properties of ILs depending on the number and length, as well as the alkyl chain branching. Thus, ILs composed of cations and anions in Table S1 can cover common hydrophobic and hydrophilic ILs. Therefore, our dataset is suitable for extracting essential cytotoxicity regardless of the limited search space. We performed regression by attaching several features to the candidates. Three physicochemical properties, i.e. refractive index, density, and viscosity, were collected for each candidate under standard conditions (298.15 K, 100 kPa) from the National Institute of Standards and Technology (NIST) database (NIST Ionic Liquids Database (SRD#147) ILthermo (v2.0)) [36] considering the database covers more than 70%–80% of the above 271 ILs for these values. We adopted values that were close to the standard conditions for some ILs for which no data were available under standard conditions. Missing data were imputed using the average feature values. In addition to these physicochemical properties, by using the Gaussian software, we used first-principles calculations based on density functional theory (DFT) to estimate the following eight molecular properties related to the cation and anion structures: the highest occupied molecular orbital (HOMO), lowest unoccupied molecular orbital (LUMO), dipole, and volume of cations and anions (Table 2). Gaussian program [37] with B3LYP functional [38] and cc-pVDZ basis set [39] was used. These features were utilized to predict the viability of hMSCs at 1 and 24 h as target features using a combination of regression and experimental synthesis. Approximately one-fifth of the search space was used as training data because the viability values were experimentally determined.

**Table 2.** Features used in this study.

	Physicochemical properties			DFT^†^ calculation properties								Target features	
Features	Refractive index	Density	Viscosity	Cation HOMO^†^	Cation LUMO^†^	Cation dipole	Cation volume	Anion HOMO	Anion LUMO	Anion dipole	Anion volume	1 h viability	24 h viability

^†^DFT: Density functional theory, HOMO: Highest occupied molecular orbital, LUMO: lowest unoccupied molecular orbital.

### 4.2. Synthesizing ILs

Among the 271 IL candidates, 51 ILs were selected based on their commercial availability and their precursors. The synthetic procedure for ILs commonly involves the ion exchange reaction of the cation precursor and the corresponding lithium salt as an anion precursor. The synthesis of triethyldodecylammonium trifluoromethylsulfonylimide (N22212-TFSI, IL131) is presented as a representative example. First, dodecyltriethylammonium bromide (N22212-Br) was prepared by reacting 6.14 mL (44.1 mmol) of triethylamine (Kanto Chemical, Japan) with 10.0 g (40.1 mmol) of bromododecane (TCI, Japan). The quaternization reaction, according to the S_N_2 mechanism, took place in acetonitrile (200 mL) at 80°C for 24 h. Repeated recrystallization of the obtained pale yellow solid from ethyl acetate yielded N22212-Br as a white solid crystal with a 69% yield. The second step involved 8.00 g (22.8 mmol) of dodecyltriethylammonium bromide as a cation precursor and a slightly excess amount (7.21 g; 25.1 mmol) of lithium trifluoromethanesulfonylimide (Li-TFSI, Solvay Japan) mixed in 30 mL of ethanol. The reaction was performed at 80°C for 15 h. After evaporation, the reaction mixture was washed with water to remove unreacted water-miscible impurities until no residual bromide anions were detected upon AgNO_3_ addition (Kanto Chemical). All ILs were dried under vacuum at 120°C for 15 h (yield: 80%). These ILs were subsequently used for the cell viability test.

### 4.3. Cell viability test

The hMSCs were purchased from PromoCell (Germany) and cultured in basal growth medium supplemented with MSC growth supplements, l-glutamine, gentamycin sulfate (30 mg·mL^−1^), and amphotericin-B (15 ng·mL^−1^). The cells were incubated in 5% CO_2_ at 37°C. Passages between 3 and 5 were used for the cell viability experiment. Briefly, we defined cell viability as cell metabolism after culturing in a medium saturated with ILs. This idea is based in accordance with ISO 10993–5, ‘Biological evaluation of medical devices Part 5: Tests for *in vitro* cytotoxicity’. We introduced 30 mL of each IL sample sterilized via UV irradiation and 200 mL of growth medium for hMSCs into microtubes. The emulsions were obtained by vigorous shaking with a vortex mixer for 1 min, with the medium and hydrophobic ILs as the continuous and dispersion phases, respectively. When hydrophilic ILs were used, the mixtures became completely homogeneous. The emulsions were subsequently centrifuged for 1 min to obtain IL-saturated medium lysates or IL emulsions. The ionic liquid-saturated medium lysate, emulsion, or hydrophilic ionic liquid-dissolved culture medium obtained via the above procedure (200 μL) was introduced to the hMSCs previously seeded in 96-well dishes and maintained at 80% confluency. The MTS ([3-(4,5-dimethylthiazol-2-yl)-5-(3carboxymethoxyphenyl)-2-(4-sulfophenyl)-2 *H*-tetrazolium) assay (Dojindo, Japan) was subsequently performed according to the manufacturer’s instructions after 1 and 24 h. The absorbance obtained from the MTS assay was defined as cell viability, which is the ratio of the absorbance of each sample divided by the absorbance of the culture medium in the absence of ILs.

### 4.4. Regression method

The Auto ML Python package Pycaret [40] was used to perform a regression analysis to predict the 1 h and 24 h viability values for the ILs. The training dataset was separated into training and testing subsets, and several regression models were evaluated using a five-fold validation. The gradient-boosting regression model [41] performed best in both cases at 1 and 24 h. The hyperparameters of the models were optimized using scikit-optimize [42],which is a sequential model-based optimization library. Regression analysis was performed to predict IL viability in the search space using the training data of the 51 ILs. We plotted a heat map for the Pearson correlation coefficient to understand the relationship between the different features of the IL and its cytotoxicity [43].

### 4.5. Procedure for cell seeding at the pristine and recycled IL interface

IL280 (N2666-TFSI, 250 μL) was placed into a circular glass bottom concave of a 12-well glass-bottom cell culture dish (MatTek, U.S.A.). The glass-bottom dish was subsequently subjected to ozone plasma treatment of the hydrophilized plastic surface surrounding the glass bottom. A 6 mg·mL^−1^ solution of fibronectin (Corning, U.S.A.) in phosphate-buffered saline (PBS; 2 mL) was covered over the IL phase followed by incubation in 5% CO_2_ at 37°C for 2 h. Subsequently, the fibronectin solution on the IL was removed by repeated rinsing at least four times with an hMSC growth medium. The hMSCs were seeded at a density of 1 × 10^4^ cells well^−1^. Bright-field images of the hMSCs attached to the IL interface were obtained after culturing for 24 h using an Olympus IX-81 microscope (Japan) equipped with a cooled CCD camera (MD695, Molecular Devices, U.S.A.). After observing the hMSC, IL280 was recovered according to the following procedure: the IL280 phase used for hMSC culturing was gently aspirated, and the centrifuge tube was collected, washed with water, and centrifuged. The IL280 phase was washed with water five times and was finally subjected to vacuum drying at 100°C for 15 h. The obtained recycled IL280 was used for interface cell culture according to the same procedure used for the pristine ILs.

### 4.6. Fluorescence observation of hMSC on IL interface

To clearly observe cell spreading morphology, hMSCs were transfected with the lifeact-GFP plasmid by electroporation. hMSC (2.0 × 10^5^ cells) were mixed with the plasmid solution (200 μg) in P1 primary cell solution (Lonza, Switzerland), and electroporated with 4D-Nucleofector (Lonza) equipped with EW104. The fluorescence images were obtained with an Olympus BX-51 microscope equipped with a LUMPlanLN 40× lens (Olympus) and the MD695 camera by using the Olympus NIBA3 filter setting.

### 4.7. Statistical analysis and reproducibility

Cell viability results were evaluated based on the average and standard deviations of the MTS assay data from three different wells. Statistical differences were evaluated based on the Student’s *t*-test.

## Supplementary Material

Supplemental Material
