# Supplementary material for: Data-driven optimization of the in silico design of ionic liquids as interfacial cell culture fluids
Source: Sci Technol Adv Mater. 2024 Oct 21;25(1):2418287. doi: 10.1080/14686996.2024.2418287 (PMC11559030; doi:10.1080/14686996.2024.2418287)
Supplement: Supplemental Material [file TSTA_A_2418287_SM3674.docx]

Supporting Information

**Data-driven Optimization of the *in silico* Design of Ionic Liquids as**

**Interfacial Cell Culture Fluids**

*Jun Nakanishi,^*^ Takeshi Ueki, Sae Dieb, Hidenori Noguchi, Shota Yamamoto, and Keitaro Sodeyama^*^*

**Figure S1** Cell adhesion test on IL280 after trice recycling

# Table S1 Dataset

Sheet 1: SMILES data

Sheet 2: DFT calculation data

Sheet 3: Viability test

Sheet 4: Predicted viability

# Figure S1


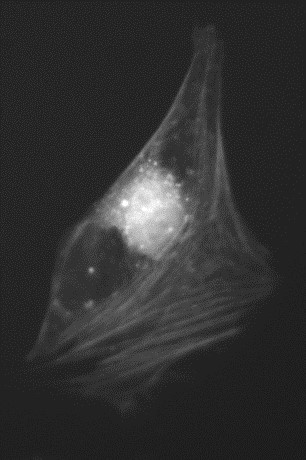

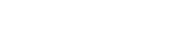


**25**

**μ**

**m**

**Figure S1** Cell adhesion test on IL280 after trice recycling. IL280 was exposed to the culture medium in the cell culture conditions (37°C, 5% CO_2_) overnight, followed by washing with water and heat-sterilization. A representative image of a hMSC expressing lifeact-GFP, attached at the IL280 interface after three cycles of recycling.

# Table S1. Dataset

Sheet 1: SMILES data

**IL**

**numb** cation name cation SMILES anion name anion SMILES **er**

| **IL001** | C2mim | CCN1C=C[N+](C)=C1 | TFSI | O=S([N-]S(=O)(C(F)(F)F)=O)(C(F)(F)F)=O |
| --- | --- | --- | --- | --- |
| **IL002** | C4mim | CCCCN1C=C[N+](C)=C1 | TFSI | O=S([N-]S(=O)(C(F)(F)F)=O)(C(F)(F)F)=O |
| **IL003** | C6mim | CCCCCCN1C=C[N+](C)=C1 | NTf2 | O=S([N-]S(=O)(C(F)(F)F)=O)(C(F)(F)F)=O |
| **IL004** | C8mim | CCCCCCCCN1C=C[N+](C)=C1 | TFSI | O=S([N-]S(=O)(C(F)(F)F)=O)(C(F)(F)F)=O |
| **IL005** | C10mim | CCCCCCCCCCN1C=C[N+](C)=C1 | TFSI | O=S([N-]S(=O)(C(F)(F)F)=O)(C(F)(F)F)=O |
| **IL006** | C12mim | CCCCCCCCCCCCN1C=C[N+](C)=C1 | TFSI | O=S([N-]S(=O)(C(F)(F)F)=O)(C(F)(F)F)=O |
| **IL007** | C4mim | CCCCN1C=C[N+](C)=C1 | NFSI | O=S([N-]S(=O)(C(F)(C(F)(C(F)(C(F)(F)F)F)F)F)=O)(C(F)(C(F)(C(F)(  C(F)(F)F)F)F)F)=O |
| **IL008** | C6mim | CCCCCCN1C=C[N+](C)=C1 | NFSI | O=S([N-]S(=O)(C(F)(C(F)(C(F)(C(F)(F)F)F)F)F)=O)(C(F)(C(F)(C(F)(  C(F)(F)F)F)F)F)=O |
| **IL009** | C8mim | CCCCCCCCN1C=C[N+](C)=C1 | NFSI | O=S([N-]S(=O)(C(F)(C(F)(C(F)(C(F)(F)F)F)F)F)=O)(C(F)(C(F)(C(F)(  C(F)(F)F)F)F)F)=O |
| **IL010** | C4Py | CCCC[n+]1ccccc1 | TFSI | O=S([N-]S(=O)(C(F)(F)F)=O)(C(F)(F)F)=O |
| **IL011** | N14Pyrr | C[N+]1(CCCC)CCCC1 | TFSI | O=S([N-]S(=O)(C(F)(F)F)=O)(C(F)(F)F)=O |
| **IL012** | N14Pipe | C[N+]1(CCCC)CCCCC1 | TFSI | O=S([N-]S(=O)(C(F)(F)F)=O)(C(F)(F)F)=O |
| **IL013** | C6Py | CCCCCC[n+]1ccccc1 | TFSI | O=S([N-]S(=O)(C(F)(F)F)=O)(C(F)(F)F)=O |
| **IL014** | N13Pipe | C[N+]1(CCC)CCCCC1 | TFSI | O=S([N-]S(=O)(C(F)(F)F)=O)(C(F)(F)F)=O |
| **IL015** | C4mim | CCCCN1C=C[N+](C)=C1 | HSO4 | OS([O-])(=O)=O |
| **IL016** | C3mim | CCCN1C=C[N+](C)=C1 | BF4 | F[B-](F)(F)F |
| **IL017** | C2mim | CCN1C=C[N+](C)=C1 | acetate | CC([O-])=O |
| **IL018** | C4mim | CCCCN1C=C[N+](C)=C1 | acetate | CC([O-])=O |
| **IL019** | C2mim | CCN1C=C[N+](C)=C1 | CF3COO | [O-]C(C(F)(F)F)=O |
| **IL020** | C2mim | CCN1C=C[N+](C)=C1 | C8-sulfate | [O-]S(=O)(OCCCCCCCC)=O |
| **IL021** | C4mim | CCCCN1C=C[N+](C)=C1 | DCA | N#C[N-]C#N |
| **IL022** | C4mim | CCCCN1C=C[N+](C)=C1 | TCN | S=C=[N-] |
| **IL023** | C6mim | CCCCCCN1C=C[N+](C)=C1 | P3C2F5 | F[P-](F)(F)(C(F)(F)C(F)(F)F)(C(F)(F)C(F)(F)F)C(F)(F)C(F)(F)F |
| **IL024** | C2mim | CCN1C=C[N+](C)=C1 | MePho | [O-]P([H])(OC)=O |
| **IL025** | C10mim | CCCCCCCCCCN1C=C[N+](C)=C1 | Br | [Br-] |
| **IL026** | C8mim | CCCCCCCCN1C=C[N+](C)=C1 | Cl | [Cl-] |
| **IL027** | C8mim | CCCCCCCCN1C=C[N+](C)=C1 | Br | [Br-] |
| **IL028** | C6mim | CCCCCCN1C=C[N+](C)=C1 | Br | [Br-] |
| **IL029** | P2225 | CC[P+](CC)(CC)CCCCC | TFSI | O=S([N-]S(=O)(C(F)(F)F)=O)(C(F)(F)F)=O |

| **IL030** | P222(C1O) | CC[P+](CC)(CC)COC | NTf2 | O=S([N-]S(=O)(C(F)(F)F)=O)(C(F)(F)F)=O |
| --- | --- | --- | --- | --- |
| **IL031** | P222(C2O) | CC[P+](CC)(CC)CCOC | NTf2 | O=S([N-]S(=O)(C(F)(F)F)=O)(C(F)(F)F)=O |
| **IL032** | P2228 | CC[P+](CC)(CC)CCCCCCCC | TFSI | O=S([N-]S(=O)(C(F)(F)F)=O)(C(F)(F)F)=O |
| **IL033** | P4441 | C[P+](CCCC)(CCCC)CCCC | TFSI | O=S([N-]S(=O)(C(F)(F)F)=O)(C(F)(F)F)=O |
| **IL034** | P66614 | CCCCCC[P+](CCCCCC)(CCCCCCCCCCCCCC)C  CCCCC | FSI | FS([N-]S(F)(=O)=O)(=O)=O |
| **IL035** | P66614 | CCCCCC[P+](CCCCCC)(CCCCCCCCCCCCCC)C  CCCCC | TFSI | O=S([N-]S(=O)(C(F)(F)F)=O)(C(F)(F)F)=O |
| **IL036** | P66614 | CCCCCC[P+](CCCCCC)(CCCCCCCCCCCCCC)C  CCCCC | CTFSI | O=S([N-]S1(=O)=O)(C(F)(F)C(F)(F)C1(F)F)=O |
| **IL037** | P66614 | CCCCCC[P+](CCCCCC)(CCCCCCCCCCCCCC)C  CCCCC | BETI | O=S([N-]S(=O)(C(F)(C(F)(F)F)F)=O)(C(F)(C(F)(F)F)F)=O |
| **IL038** | P66614 | CCCCCC[P+](CCCCCC)(CCCCCCCCCCCCCC)C  CCCCC | NFSI | O=S([N-]S(=O)(C(F)(C(F)(C(F)(C(F)(F)F)F)F)F)=O)(C(F)(C(F)(C(F)(  C(F)(F)F)F)F)F)=O |
| **IL039** | P8888 | CCCCCCCC[P+](CCCCCCCC)(CCCCCCCC)CCC  CCCCC | NFSI | O=S([N-]S(=O)(C(F)(C(F)(C(F)(C(F)(F)F)F)F)F)=O)(C(F)(C(F)(C(F)(  C(F)(F)F)F)F)F)=O |
| **IL040** | P8888 | CCCCCCCC[P+](CCCCCCCC)(CCCCCCCC)CCC  CCCCC | TFSI | O=S([N-]S(=O)(C(F)(F)F)=O)(C(F)(F)F)=O |
| **IL041** | P8888 | CCCCCCCC[P+](CCCCCCCC)(CCCCCCCC)CCC  CCCCC | CTFSI | O=S([N-]S1(=O)=O)(C(F)(F)C(F)(F)C1(F)F)=O |
| **IL042** | P8888 | CCCCCCCC[P+](CCCCCCCC)(CCCCCCCC)CCC  CCCCC | BETI | O=S([N-]S(=O)(C(F)(C(F)(F)F)F)=O)(C(F)(C(F)(F)F)F)=O |
| **IL043** | P66614 | CCCCCC[P+](CCCCCC)(CCCCCCCCCCCCCC)C  CCCCC | Br | [Br-] |
| **IL044** | C2mim | CCN1C=C[N+](C)=C1 | BF4 | F[B-](F)(F)F |
| **IL045** | C4mim | CCCCN1C=C[N+](C)=C1 | BF4 | F[B-](F)(F)F |
| **IL046** | C2dmim | CCN1C=C[N+](C)=C1(C) | BF4 | F[B-](F)(F)F |
| **IL047** | C2dmim | CCN1C=C[N+](C)=C1(C) | TFSI | O=S([N-]S(=O)(C(F)(F)F)=O)(C(F)(F)F)=O |
| **IL048** | C8mim | CCCCCCCCN1C=C[N+](C)=C1 | BF4 | F[B-](F)(F)F |
| **IL049** | C2mimOH | C[N+]1=CN(CCO)C=C1 | BF4 | F[B-](F)(F)F |
| **IL050** | C2mimOH | C[N+]1=CN(CCO)C=C1 | TFSI | O=S([N-]S(=O)(C(F)(F)F)=O)(C(F)(F)F)=O |
| **IL051** | DEME | C[N+](CC)(CCOC)CC | BF4 | F[B-](F)(F)F |
| **IL052** | N14Pyrr | C[N+]1(CCCC)CCCC1 | BF4 | F[B-](F)(F)F |
| **IL053** | C2mim | CCN1C=C[N+](C)=C1 | HSO4 | OS([O-])(=O)=O |
| **IL054** | C0mim | N1C=C[N+](C)=C1 | TFSI | O=S([N-]S(=O)(C(F)(F)F)=O)(C(F)(F)F)=O |
| **IL055** | dema | CC[NH+](C)CC | TfO | O=S(C(F)(F)F)([O-])=O |
| **IL056** | C4mim | CCCCN1C=C[N+](C)=C1 | I | [I-] |
| **IL057** | C4mim | CCCCN1C=C[N+](C)=C1 | PF6 | F[P-](F)(F)(F)(F)F |
| **IL058** | C6mim | CCCCCCN1C=C[N+](C)=C1 | PF6 | F[P-](F)(F)(F)(F)F |
| **IL059** | C8mim | CCCCCCCCN1C=C[N+](C)=C1 | PF6 | F[P-](F)(F)(F)(F)F |
| **IL060** | C4Py | CCCC[n+]1ccccc1 | BF4 | F[B-](F)(F)F |
| **IL061** | C3Py | CCC[n+]1ccccc1 | BF4 | F[B-](F)(F)F |
| **IL062** | 2-MeC4Py | CCCC[n+]1c(C)cccc1 | BF4 | F[B-](F)(F)F |

| **IL063** | 4-MeC4Py | CCCC[n+]1ccc(C)cc1 | BF4 | F[B-](F)(F)F |
| --- | --- | --- | --- | --- |
| **IL064** | 3-MeC8Py | CCCCCCCC[n+]1cc(C)ccc1 | BF4 | F[B-](F)(F)F |
| **IL065** | C5mim | CCCCCN1C=C[N+](C)=C1 | BF4 | F[B-](F)(F)F |
| **IL066** | C4mimCN | N#CCCCN1C=C[N+](C)=C1 | BF4 | F[B-](F)(F)F |
| **IL067** | C4dmim | CCCCN1C=C[N+](C)=C1(C) | BF4 | F[B-](F)(F)F |
| **IL068** | C6Py | CCCCCC[n+]1ccccc1 | BF4 | F[B-](F)(F)F |
| **IL069** | 1-Ph 3,5-Me  2-Pen  Pyrazo | CCCCCN1[N+](C2=CC=CC=C2)=C(C)C=C1C | BF4 | F[B-](F)(F)F |
| **IL070** | 1-Ph 3,5-Me  2-Hex  Pyrazo | CC1=CC(C)=[N+](C2=CC=CC=C2)N1CCCCCC | BF4 | F[B-](F)(F)F |
| **IL071** | C5mim | CCCCCN1C=C[N+](C)=C1 | PF6 | F[P-](F)(F)(F)(F)F |
| **IL072** | C9mim | CCCCCCCCCN1C=C[N+](C)=C1 | PF6 | F[P-](F)(F)(F)(F)F |
| **IL073** | C2Py | CC[n+]1ccccc1 | TFSI | O=S([N-]S(=O)(C(F)(F)F)=O)(C(F)(F)F)=O |
| **IL074** | N1113 | C[N+](CCC)(C)C | TFSI | O=S([N-]S(=O)(C(F)(F)F)=O)(C(F)(F)F)=O |
| **IL075** | N1114 | C[N+](CCCC)(C)C | TFSI | O=S([N-]S(=O)(C(F)(F)F)=O)(C(F)(F)F)=O |
| **IL076** | 2-MeC2Py | CC[n+]1c(C)cccc1 | TFSI | O=S([N-]S(=O)(C(F)(F)F)=O)(C(F)(F)F)=O |
| **IL077** | Ciso4mim | CC(C)CN1C=C[N+](C)=C1 | TFSI | O=S([N-]S(=O)(C(F)(F)F)=O)(C(F)(F)F)=O |
| **IL078** | C3mim | CCCN1C=C[N+](C)=C1 | TFSI | O=S([N-]S(=O)(C(F)(F)F)=O)(C(F)(F)F)=O |
| **IL079** | N13Pyrr | C[N+]1(CCC)CCCC1 | TFSI | O=S([N-]S(=O)(C(F)(F)F)=O)(C(F)(F)F)=O |
| **IL080** | N2225 | CC[N+](CC)(CCCCC)CC | TFSI | O=S([N-]S(=O)(C(F)(F)F)=O)(C(F)(F)F)=O |
| **IL081** | 3-MeC3Py | CCC[n+]1cc(C)ccc1 | TFSI | O=S([N-]S(=O)(C(F)(F)F)=O)(C(F)(F)F)=O |
| **IL082** | 3-MeC4Py | CCCC[n+]1cc(C)ccc1 | TFSI | O=S([N-]S(=O)(C(F)(F)F)=O)(C(F)(F)F)=O |
| **IL083** | 4-MeC4Py | CCCC[n+]1ccc(C)cc1 | TFSI | O=S([N-]S(=O)(C(F)(F)F)=O)(C(F)(F)F)=O |
| **IL084** | 2-MeC3Py | CCC[N+]1=C(C)C=CC=C1 | TFSI | O=S([N-]S(=O)(C(F)(F)F)=O)(C(F)(F)F)=O |
| **IL085** | NH2-Im-C4-  Im-NH2 | NC(C[N+]1=CN(CCCCN2C=C[N+](CC(N)=O)=  C2)C=C1)=O | TFSI | O=S([N-]S(=O)(C(F)(F)F)=O)(C(F)(F)F)=O |
| **IL086** | C10Py | CCCCCCCCCC[n+]1ccccc1 | TFSI | O=S([N-]S(=O)(C(F)(F)F)=O)(C(F)(F)F)=O |
| **IL087** | N1iso4Pipe | C[N+]1(CC(C)C)CCCCC1 | TFSI | O=S([N-]S(=O)(C(F)(F)F)=O)(C(F)(F)F)=O |
| **IL088** | 2-Pe3,5-  MeC2Py | CC[n+]1c(CCCCC)c(C)cc(C)c1 | TFSI | O=S([N-]S(=O)(C(F)(F)F)=O)(C(F)(F)F)=O |
| **IL089** | 2-Pe3,5-  MeC4Py | CCCC[n+]1c(CCCCC)c(C)cc(C)c1 | TFSI | O=S([N-]S(=O)(C(F)(F)F)=O)(C(F)(F)F)=O |
| **IL090** | 2,3,5-  MeC6Py | CCCCCC[n+]1c(C)c(C)cc(C)c1 | TFSI | O=S([N-]S(=O)(C(F)(F)F)=O)(C(F)(F)F)=O |
| **IL091** | 2-Et3,5-  MeC6Py | CCCCCC[n+]1c(CC)c(C)cc(C)c1 | TFSI | O=S([N-]S(=O)(C(F)(F)F)=O)(C(F)(F)F)=O |
| **IL092** | 2-Pe3,5-  MeC6Py | CCCCCC[n+]1c(CCCCC)c(C)cc(C)c1 | TFSI | O=S([N-]S(=O)(C(F)(F)F)=O)(C(F)(F)F)=O |
| **IL093** | C12Py | CCCCCCCCCCCC[n+]1ccccc1 | TFSI | O=S([N-]S(=O)(C(F)(F)F)=O)(C(F)(F)F)=O |
| **IL094** | C14mim | CCCCCCCCCCCCCCN1C=C[N+](C)=C1 | TFSI | O=S([N-]S(=O)(C(F)(F)F)=O)(C(F)(F)F)=O |

| **IL095** | N1iso4Pyrr | C[N+]1(CC(C)C)CCCC1 | TFSI | O=S([N-]S(=O)(C(F)(F)F)=O)(C(F)(F)F)=O |
| --- | --- | --- | --- | --- |
| **IL096** | 3-  MeCiso4Py | CC(C)C[N+]1=CC(C)=CC=C1 | TFSI | O=S([N-]S(=O)(C(F)(F)F)=O)(C(F)(F)F)=O |
| **IL097** | C44im | CCCCN1C=[N+](CCCC)C=C1 | TFSI | O=S([N-]S(=O)(C(F)(F)F)=O)(C(F)(F)F)=O |
| **IL098** | 3,5-MeC6Py | CC1=CC(C)=C[N+](CCCCCC)=C1 | TFSI | O=S([N-]S(=O)(C(F)(F)F)=O)(C(F)(F)F)=O |
| **IL099** | N2228 | CC[N+](CC)(CCCCCCCC)CC | TFSI | O=S([N-]S(=O)(C(F)(F)F)=O)(C(F)(F)F)=O |
| **IL100** | C3CN2O2im | N#CCC[N+]1=CN(CCOCC)C=C1 | TFSI | O=S([N-]S(=O)(C(F)(F)F)=O)(C(F)(F)F)=O |
| **IL101** | 3-MeC4Py | CCCC[n+]1cc(C)ccc1 | DCA | N#C[N-]C#N |
| **IL102** | P66614 | CCCCCC[P+](CCCCCC)(CCCCCCCCCCCCCC)C  CCCCC | DCA | N#C[N-]C#N |
| **IL103** | 3-MeC2Py | CC[n+]1cc(C)ccc1 | FSI | FS([N-]S(F)(=O)=O)(=O)=O |
| **IL104** | C6Py | CCCCCC[n+]1ccccc1 | FSI | FS([N-]S(F)(=O)=O)(=O)=O |
| **IL105** | C4Py | CCCC[n+]1ccccc1 | FSI | FS([N-]S(F)(=O)=O)(=O)=O |
| **IL106** | C2Py | CC[n+]1ccccc1 | FSI | FS([N-]S(F)(=O)=O)(=O)=O |
| **IL107** | N13Pyrr | C[N+]1(CCC)CCCC1 | FSI | FS([N-]S(F)(=O)=O)(=O)=O |
| **IL108** | C2mim | CCN1C=C[N+](C)=C1 | FSI | FS([N-]S(F)(=O)=O)(=O)=O |
| **IL109** | N14Pyrr | C[N+]1(CCCC)CCCC1 | FSI | FS([N-]S(F)(=O)=O)(=O)=O |
| **IL110** | N14Pyrr | C[N+]1(CCCC)CCCC1 | DCA | N#C[N-]C#N |
| **IL111** | C6mim | CCCCCCN1C=C[N+](C)=C1 | DCA | N#C[N-]C#N |
| **IL112** | Choline | OCC[N+](C)(C)C | NFSI | O=S([N-]S(=O)(C(F)(C(F)(C(F)(C(F)(F)F)F)F)F)=O)(C(F)(C(F)(C(F)(  C(F)(F)F)F)F)F)=O |
| **IL113** | C2mC1O | CC[N+]1=CN(CCOC)C=C1 | DCA | N#C[N-]C#N |
| **IL114** | C18dienmim | C[N+]1=CN(CCCCCCCC/C=C/C/C=C\CCCCC)  C=C1 | TFSI | O=S([N-]S(=O)(C(F)(F)F)=O)(C(F)(F)F)=O |
| **IL115** | C18monoen mim | C[N+]1=CN(CCCCCCCC/C=C/CCCCCCCC)C=  C1 | TFSI | O=S([N-]S(=O)(C(F)(F)F)=O)(C(F)(F)F)=O |
| **IL116** | C4Py | CCCC[n+]1ccccc1 | DCA | N#C[N-]C#N |
| **IL117** | C3Py | CCC[n+]1ccccc1 | DCA | N#C[N-]C#N |
| **IL118** | C6Py | CCCCCC[n+]1ccccc1 | DCA | N#C[N-]C#N |
| **IL119** | C3CN2O2im | N#CCC[N+]1=CN(CCOCC)C=C1 | DCA | N#C[N-]C#N |
| **IL120** | C4CNmim | C[N+]1=CN(CCCC#N)C=C1 | TFSI | O=S([N-]S(=O)(C(F)(F)F)=O)(C(F)(F)F)=O |
| **IL121** | N1888 | C[N+](CCCCCCCC)(CCCCCCCC)CCCCCCCC | TFSI | O=S([N-]S(=O)(C(F)(F)F)=O)(C(F)(F)F)=O |
| **IL122** | N110 Pyrr | C[N+]1(CCCCCCCCCC)CCCC1 | TFSI | O=S([N-]S(=O)(C(F)(F)F)=O)(C(F)(F)F)=O |
| **IL123** | N113iso3 | C[N+](C)(C(C)C)CCC | TFSI | O=S([N-]S(=O)(C(F)(F)F)=O)(C(F)(F)F)=O |
| **IL124** | N12OH Pyrr | C[N+]1(CCO)CCCC1 | TFSI | O=S([N-]S(=O)(C(F)(F)F)=O)(C(F)(F)F)=O |
| **IL125** | N116iso3 | C[N+](C)(C(C)C)CCCCCC | TFSI | O=S([N-]S(=O)(C(F)(F)F)=O)(C(F)(F)F)=O |
| **IL126** | N1110iso3 | C[N+](C)(C(C)C)CCCCCCCCCC | TFSI | O=S([N-]S(=O)(C(F)(F)F)=O)(C(F)(F)F)=O |
| **IL127** | N112OHiso3 | C[N+](C)(C(C)C)CCO | TFSI | O=S([N-]S(=O)(C(F)(F)F)=O)(C(F)(F)F)=O |
| **IL128** | N1(OH)2  Pyrr | C[N+]1(CC(O)CO)CCCC1 | TFSI | O=S([N-]S(=O)(C(F)(F)F)=O)(C(F)(F)F)=O |

| **IL129** | N11114 | C[N+](C)(CCCCCCCCCCCCCC)C | TFSI | O=S([N-]S(=O)(C(F)(F)F)=O)(C(F)(F)F)=O |
| --- | --- | --- | --- | --- |
| **IL130** | N22210 | CC[N+](CC)(CCCCCCCCCC)CC | TFSI | O=S([N-]S(=O)(C(F)(F)F)=O)(C(F)(F)F)=O |
| **IL131** | N22212 | CC[N+](CC)(CCCCCCCCCCCC)CC | TFSI | O=S([N-]S(=O)(C(F)(F)F)=O)(C(F)(F)F)=O |
| **IL132** | C6Diaza | CCCCCC[N+]1(CC2)CCN2CC1 | TFSI | O=S([N-]S(=O)(C(F)(F)F)=O)(C(F)(F)F)=O |
| **IL133** | 3,5-MeC4Py | CC1=CC(C)=C[N+](CCCC)=C1 | TFSI | O=S([N-]S(=O)(C(F)(F)F)=O)(C(F)(F)F)=O |
| **IL134** | 2-Me,5-  EtC4Py | CC1=[N+](CCCC)C=C(CC)C=C1 | TFSI | O=S([N-]S(=O)(C(F)(F)F)=O)(C(F)(F)F)=O |
| **IL135** | 2,3,5-  MeC4Py | CC1=[N+](CCCC)C=C(C)C=C1C | TFSI | O=S([N-]S(=O)(C(F)(F)F)=O)(C(F)(F)F)=O |
| **IL136** | 3,5-MeC8Py | CC1=CC(C)=C[N+](CCCCCCCC)=C1 | TFSI | O=S([N-]S(=O)(C(F)(F)F)=O)(C(F)(F)F)=O |
| **IL137** | 2,3-MeC8Py | CCCCCCCC[N+]1=CC=CC(C)=C1C | TFSI | O=S([N-]S(=O)(C(F)(F)F)=O)(C(F)(F)F)=O |
| **IL138** | 2,3,5-  MeC8Py | CCCCCCCC[n+]1c(C)c(C)cc(C)c1 | TFSI | O=S([N-]S(=O)(C(F)(F)F)=O)(C(F)(F)F)=O |
| **IL139** | 2,3-MeC4Py | CCCC[N+]1=CC=CC(C)=C1C | TFSI | O=S([N-]S(=O)(C(F)(F)F)=O)(C(F)(F)F)=O |
| **IL140** | 2,4-MeC6Py | CC1=CC(C)=CC=[N+]1CCCCCC | TFSI | O=S([N-]S(=O)(C(F)(F)F)=O)(C(F)(F)F)=O |
| **IL141** | C4CNdmim | CN1C=C[N+](CCCC#N)=C1C | TFSI | O=S([N-]S(=O)(C(F)(F)F)=O)(C(F)(F)F)=O |
| **IL142** | C4CNdmim | CN1C=C[N+](CCCC#N)=C1C | DCA | N#C[N-]C#N |
| **IL143** | C4PyCN | N#CCCC[N+]1=CC=CC=C1 | TFSI | O=S([N-]S(=O)(C(F)(F)F)=O)(C(F)(F)F)=O |
| **IL144** | C4PyCN | N#CCCC[N+]1=CC=CC=C1 | DCA | N#C[N-]C#N |
| **IL145** | C4mimCN | N#CCCCN1C=C[N+](C)=C1 | DCA | N#C[N-]C#N |
| **IL146** | C8mim | CCCCCCCCN1C=C[N+](C)=C1 | TCN | S=C=[N-] |
| **IL147** | C6mim | CCCCCCN1C=C[N+](C)=C1 | TCN | S=C=[N-] |
| **IL148** | 3,5-MeC8Py | CC1=CC(C)=C[N+](CCCCCCCC)=C1 | TCN | S=C=[N-] |
| **IL149** | 2,3-MeC8Py | CCCCCCCC[N+]1=CC=CC(C)=C1C | TCN | S=C=[N-] |
| **IL150** | P4442 | CCCC[P+](CCCC)(CC)CCCC | diEt-  Phosphate | [O-]P(OCC)(OCC)=O |
| **IL151** | C2mim | CCN1C=C[N+](C)=C1 | P3C2F5 | F[P-](F)(F)(C(F)(F)C(F)(F)F)(C(F)(F)C(F)(F)F)C(F)(F)C(F)(F)F |
| **IL152** | C2mim | CCN1C=C[N+](C)=C1 | diMe-  Phosphate | [O-]P(OC)(OC)=O |
| **IL153** | C2mim | CCN1C=C[N+](C)=C1 | diEt-  Phosphate | [O-]P(OCC)(OCC)=O |
| **IL154** | P4441 | C[P+](CCCC)(CCCC)CCCC | Me-sulfate | [O-]S(=O)(OC)=O |
| **IL155** | C2mim | CCN1C=C[N+](C)=C1 | Et-sulfate | [O-]S(=O)(OCC)=O |
| **IL156** | C4mim | CCCCN1C=C[N+](C)=C1 | C8-sulfate | [O-]S(=O)(OCCCCCCCC)=O |
| **IL157** | C1mim | CN1C=C[N+](C)=C1 | Me-sulfate | [O-]S(=O)(OC)=O |
| **IL158** | C4mim | CCCCN1C=C[N+](C)=C1 | Me-sulfate | [O-]S(=O)(OC)=O |
| **IL159** | L-Ala | C[C@H]([NH3+])C(OC(C)C)=O | C12-sulfate | [O-]S(=O)(OCCCCCCCCCCCC)=O |
| **IL160** | 2-iso4 Pyrr | O=C(OCC(C)C)[C@H]1[NH2+]CCC1 | C12-sulfate | [O-]S(=O)(OCCCCCCCCCCCC)=O |
| **IL161** | 1-iso3 3-Me aminium | CC([C@H]([NH3+])C(OC(C)C)=O)C | C12-sulfate | [O-]S(=O)(OCCCCCCCCCCCC)=O |
| **IL162** | 2-iso3 Pyrr | O=C(OC(C)C)[C@@H]1CCC[NH2+]1 | C12-sulfate | [O-]S(=O)(OCCCCCCCCCCCC)=O |

| **IL163** | 1-iso4 1-Opr aminium | C[C@H]([NH3+])C(OCC(C)C)=O | C12-sulfate | [O-]S(=O)(OCCCCCCCCCCCC)=O |
| --- | --- | --- | --- | --- |
| **IL164** | 1,5-iso3 1,5dioxo5 aminium | CC(OC([C@@H]([NH3+])CCC(OC(C)C)=O)=O)  C | C12-sulfate | [O-]S(=O)(OCCCCCCCCCCCC)=O |
| **IL165** | 2-EtC1Py | C[N+]1=CC=CC=C1CC | Me-sulfate | [O-]S(=O)(OC)=O |
| **IL166** | 2-EtC2Py | CCC1=CC=CC=[N+]1CC | Et-sulfate | [O-]S(=O)(OCC)=O |
| **IL167** | C1Py | C[N+]1=CC=CC=C1 | Me-sulfate | [O-]S(=O)(OC)=O |
| **IL168** | 3-MeC1Py | C[N+]1=CC(C)=CC=C1 | Me-sulfate | [O-]S(=O)(OC)=O |
| **IL169** | C2Py | CC[n+]1ccccc1 | Et-sulfate | [O-]S(=O)(OCC)=O |
| **IL170** | C2mim | CCN1C=C[N+](C)=C1 | Me-sulfate | [O-]S(=O)(OC)=O |
| **IL171** | Cbnmim | C[N+]1=CN(CC2=CC=CC=C2)C=C1 | Me-sulfate | [O-]S(=O)(OC)=O |
| **IL172** | 3-MeC2Py | CC[n+]1cc(C)ccc1 | Et-sulfate | [O-]S(=O)(OCC)=O |
| **IL173** | N14Pyrr | C[N+]1(CCCC)CCCC1 | Me-sulfate | [O-]S(=O)(OC)=O |
| **IL174** | N2221 | C[N+](CC)(CC)CC | Me-sulfate | [O-]S(=O)(OC)=O |
| **IL175** | N12Pyrr | C[N+]1(CC)CCCC1 | Et-sulfate | [O-]S(=O)(OCC)=O |
| **IL176** | C4pro-CNim | N#CCC[N+]1=CN(CCCC)C=C1 | C12-sulfate | [O-]S(=O)(OCCCCCCCCCCCC)=O |
| **IL177** | C10pro-  CNim | N#CCC[N+]1=CN(CCCCCCCCCC)C=C1 | C12-sulfate | [O-]S(=O)(OCCCCCCCCCCCC)=O |
| **IL178** | C8pro-CNim | N#CCC[N+]1=CN(CCCCCCCC)C=C1 | C10-sulfate | [O-]S(=O)(OCCCCCCCCCC)=O |
| **IL179** | C6pro-CNim | N#CCC[N+]1=CN(CCCCCC)C=C1 | C12-sulfate | [O-]S(=O)(OCCCCCCCCCCCC)=O |
| **IL180** | C0mim | N1C=C[N+](C)=C1 | HSO4 | OS([O-])(=O)=O |
| **IL181** | C0Buim | CCCC[N+]1=CNC=C1 | HSO4 | OS([O-])(=O)=O |
| **IL182** | C2mim | CCN1C=C[N+](C)=C1 | MeOEtOEt-  sulfate | [O-]S(=O)(OCCOCCOC)=O |
| **IL183** | N24Pyrr | CC[N+]1(CCCC)CCCC1 | Et-sulfate | [O-]S(=O)(OCC)=O |
| **IL184** | C3mim | CCCN1C=C[N+](C)=C1 | Me-sulfate | [O-]S(=O)(OC)=O |
| **IL185** | C6mim | CCCCCCN1C=C[N+](C)=C1 | Et-sulfate | [O-]S(=O)(OCC)=O |
| **IL186** | C8mim | CCCCCCCCN1C=C[N+](C)=C1 | MeOEtOEt-  sulfate | [O-]S(=O)(OCCOCCOC)=O |
| **IL187** | N1(EtOH)3 | OCC[N+](CCO)(CCO)C | Me-sulfate | [O-]S(=O)(OC)=O |
| **IL188** | C2etim | CC[N+]1=CN(CC)C=C1 | Et-sulfate | [O-]S(=O)(OCC)=O |
| **IL189** | N0002 | CC[NH3+] | HSO4 | OS([O-])(=O)=O |
| **IL190** | C4mim | CCCCN1C=C[N+](C)=C1 | MeOEtOEt-  sulfate | [O-]S(=O)(OCCOCCOC)=O |
| **IL191** | N1122OH | OCC[N+](C)(CC)C | Et-sulfate | [O-]S(=O)(OCC)=O |
| **IL192** | N1124 | C[N+](CCCC)(C)CC | Et-sulfate | [O-]S(=O)(OCC)=O |
| **IL193** | N0022 | CC[NH2+]CC | HSO4 | OS([O-])(=O)=O |
| **IL194** | N0111 | C[NH+](C)C | HSO4 | OS([O-])(=O)=O |
| **IL195** | N0222 | CC[NH+](CC)CC | HSO4 | OS([O-])(=O)=O |

| **IL196** | C2Py | CC[n+]1ccccc1 | TfO | O=S(C(F)(F)F)([O-])=O |
| --- | --- | --- | --- | --- |
| **IL197** | C4Py | CCCC[n+]1ccccc1 | TfO | O=S(C(F)(F)F)([O-])=O |
| **IL198** | C2mim | CCN1C=C[N+](C)=C1 | TfO | O=S(C(F)(F)F)([O-])=O |
| **IL199** | C4mim | CCCCN1C=C[N+](C)=C1 | TfO | O=S(C(F)(F)F)([O-])=O |
| **IL200** | N14Pyrr | C[N+]1(CCCC)CCCC1 | TfO | O=S(C(F)(F)F)([O-])=O |
| **IL201** | 3-MeC4Py | CCCC[n+]1cc(C)ccc1 | TfO | O=S(C(F)(F)F)([O-])=O |
| **IL202** | C42im | CC[N+]1=CN(CCCC)C=C1 | TfO | O=S(C(F)(F)F)([O-])=O |
| **IL203** | 2,3-MeC4Py | CCCC[N+]1=CC=CC(C)=C1C | TfO | O=S(C(F)(F)F)([O-])=O |
| **IL204** | C6mim | CCCCCCN1C=C[N+](C)=C1 | TfO | O=S(C(F)(F)F)([O-])=O |
| **IL205** | C43CNim | N#CCCN1C=[N+](CCCC)C=C1 | TfO | O=S(C(F)(F)F)([O-])=O |
| **IL206** | C63CNim | N#CCCN1C=[N+](CCCCCC)C=C1 | TfO | O=S(C(F)(F)F)([O-])=O |
| **IL207** | C83CNim | N#CCCN1C=[N+](CCCCCCCC)C=C1 | TfO | O=S(C(F)(F)F)([O-])=O |
| **IL208** | 2,3-MeC8Py | CCCCCCCC[N+]1=CC=CC(C)=C1C | TfO | O=S(C(F)(F)F)([O-])=O |
| **IL209** | P66614 | CCCCCC[P+](CCCCCC)(CCCCCCCCCCCCCC)C  CCCCC | TfO | O=S(C(F)(F)F)([O-])=O |
| **IL210** | C4mim | CCCCN1C=C[N+](C)=C1 | Br | [Br-] |
| **IL211** | C43CNim | N#CCCN1C=[N+](CCCC)C=C1 | Br | [Br-] |
| **IL212** | C63CNim | N#CCCN1C=[N+](CCCCCC)C=C1 | Br | [Br-] |
| **IL213** | C83CNim | N#CCCN1C=[N+](CCCCCCCC)C=C1 | Br | [Br-] |
| **IL214** | P8888 | CCCCCCCC[P+](CCCCCCCC)(CCCCCCCC)CCC  CCCCC | Br | [Br-] |
| **IL215** | C3all3CN | N#CCCN1C=[N+](CC=C)C=C1 | Cl | [Cl-] |
| **IL216** | C2OH3CN | N#CCCN1C=[N+](CCO)C=C1 | Cl | [Cl-] |
| **IL217** | C6mim | CCCCCCN1C=C[N+](C)=C1 | Cl | [Cl-] |
| **IL218** | C6OH0im | OCCCCCC[N+]1=CN=CC1 | Cl | [Cl-] |
| **IL219** | CBnmim | C[N+]1=CN(C=C1)CC2=CC=CC=C2 | Cl | [Cl-] |
| **IL220** | C43CNim | N#CCCN1C=[N+](CCCC)C=C1 | Cl | [Cl-] |
| **IL221** | C6OHmim | OCCCCCC[N+]1=CN(C)C=C1 | Cl | [Cl-] |
| **IL222** | C63CNim | N#CCCN1C=[N+](CCCCCC)C=C1 | Cl | [Cl-] |
| **IL223** | C10mim | CCCCCCCCCCN1C=C[N+](C)=C1 | Cl | [Cl-] |
| **IL224** | C46OHim | CCCC[N+]1=CN(CCCCCCO)C=C1 | Cl | [Cl-] |
| **IL225** | C83CNim | N#CCCN1C=[N+](CCCCCCCC)C=C1 | Cl | [Cl-] |
| **IL226** | P4448 | CCCC[P+](CCCC)(CCCCCCCC)CCCC | Cl | [Cl-] |
| **IL227** | P66614 | CCCCCC[P+](CCCCCC)(CCCCCCCCCCCCCC)C  CCCCC | Cl | [Cl-] |
| **IL228** | N0002 | CC[NH3+] | acetate | CC([O-])=O |
| **IL229** | N0111 | C[NH+](C)C | acetate | CC([O-])=O |

| **IL230** | N0003 | [NH3+]CCC | acetate | CC([O-])=O |
| --- | --- | --- | --- | --- |
| **IL231** | N0002 | CC[NH3+] | glycolate | [O-]C(CO)=O |
| **IL232** | N0004 | [NH3+]CCCC | acetate | CC([O-])=O |
| **IL233** | N0022 | CC[NH2+]CC | acetate | CC([O-])=O |
| **IL234** | N0112 | C[NH+](C)CC | acetate | CC([O-])=O |
| **IL235** | N0012OH | OCC[NH2+]C | acetate | CC([O-])=O |
| **IL236** | C0mim | N1C=C[N+](C)=C1 | acetate | CC([O-])=O |
| **IL237** | N0222 | CC[NH+](CC)CC | acetate | CC([O-])=O |
| **IL238** | N1112OH | OCC[N+](C)(C)C | glycinate | NCC([O-])=O |
| **IL239** | C03CNim | N#CCC[N+]1=CNC=C1 | acetate | CC([O-])=O |
| **IL240** | C3mim | CCCN1C=C[N+](C)=C1 | acetate | CC([O-])=O |
| **IL241** | C2mim | CCN1C=C[N+](C)=C1 | ammonioace  tate | [O-]C(CN)=O |
| **IL242** | N14Pyrr | C[N+]1(CCCC)CCCC1 | acetate | CC([O-])=O |
| **IL243** | C5mim | CCCCCN1C=C[N+](C)=C1 | acetate | CC([O-])=O |
| **IL244** | C6mim | CCCCCCN1C=C[N+](C)=C1 | acetate | CC([O-])=O |
| **IL245** | CEtOEt3CN | N#CCCN1C=[N+](CCOCC)C=C1 | CF3COO | [O-]C(C(F)(F)F)=O |
| **IL246** | C4mim | CCCCN1C=C[N+](C)=C1 | ibuprofenate | CC(CC1=CC=C(C(C([O-])=O)C)C=C1)C |
| **IL247** | P66614 | CCCCCC[P+](CCCCCC)(CCCCCCCCCCCCCC)C  CCCCC | acetate | CC([O-])=O |
| **IL248** | N0002 | CC[NH3+] | NO3 | [O-][N+]([O-])=O |
| **IL249** | N0002OH | [NH3+]CCO | NO3 | [O-][N+]([O-])=O |
| **IL250** | Pyrr | [NH2+]1CCCC1 | NO3 | [O-][N+]([O-])=O |
| **IL251** | N0004 | [NH3+]CCCC | NO3 | [O-][N+]([O-])=O |
| **IL252** | C2mim | CCN1C=C[N+](C)=C1 | Me-  sulfonate | CS(=O)([O-])=O |
| **IL253** | C2mim | CCN1C=C[N+](C)=C1 | taurinate | NCCS(=O)([O-])=O |
| **IL254** | N1122OH | OCC[N+](C)(CC)C | Bu-sulfonate | O=S(CCCC)([O-])=O |
| **IL255** | C2mim | CCN1C=C[N+](C)=C1 | 4-Me Bnsulfonate | O=S([O-])(C1=CC=C(C)C=C1)=O |
| **IL256** | 1-Ph 2,3,5-  Me Pyraz | CN1[N+](C2=CC=CC=C2)=C(C)C=C1C | Me-  sulfonate | CS(=O)([O-])=O |
| **IL257** | C03CNim | N#CCC[N+]1=CNC=C1 | 4-Me Bnsulfonate | O=S([O-])(C1=CC=C(C)C=C1)=O |
| **IL258** | N1112OH | OCC[N+](C)(C)C | OHAmEt-  solfonate | O=S([O-])(CCN(CCO)CCO)=O |
| **IL259** | C2mim | CCN1C=C[N+](C)=C1 | AAMP-  sulfonate | O=S(CC(C)(C)NC(C=C)=O)([O-])=O |
| **IL260** | 1-Ph 2-Bu  3,5-Me  Pyraz | CC1=CC(C)=[N+](C2=CC=CC=C2)N1CCCC | Me-  sulfonate | CS(=O)([O-])=O |
| **IL261** | N1112OH | OCC[N+](C)(C)C | MorOH-  sulfonate | OC(CS(=O)([O-])=O)CN1CCOCC1 |
| **IL262** | C2mim | CCN1C=C[N+](C)=C1 | BETI | O=S([N-]S(=O)(C(F)(C(F)(F)F)F)=O)(C(F)(C(F)(F)F)F)=O |

| **IL263** | C2mim | CCN1C=C[N+](C)=C1 | TCN | S=C=[N-] |
| --- | --- | --- | --- | --- |
| **IL264** | C2mim | CCN1C=C[N+](C)=C1 | DCA | N#C[N-]C#N |
| **IL265** | N1112OH | OCC[N+](C)(C)C | lactate | CC(C([O-])=O)O |
| **IL266** | C2mim | CCN1C=C[N+](C)=C1 | lactate | CC(C([O-])=O)O |
| **IL267** | S122 | C[S+](CC)CC | TFSI | O=S([N-]S(=O)(C(F)(F)F)=O)(C(F)(F)F)=O |
| **IL268** | N2OH | OCC[NH3+] | HCOO | [H]C([O-])=O |
| **IL269** | Bmim | CC[N+]1=CN(CCCC)C=C1 | BF4 | F[B-](F)(F)F |
| **IL270** | C6mim | CCCCCCN1C=C[N+](C)=C1 | BF4 | F[B-](F)(F)F |
| **IL271** | C10mim | CCCCCCCCCCN1C=C[N+](C)=C1 | BF4 | F[B-](F)(F)F |
| **IL272** | Choline | OCC[N+](C)(C)C | TFSI | O=S([N-]S(=O)(C(F)(F)F)=O)(C(F)(F)F)=O |
| **IL273** | N1116 | C[N+](C)(CCCCCC)C | TFSI | O=S([N-]S(=O)(C(F)(F)F)=O)(C(F)(F)F)=O |
| **IL274** | N4441 | C[N+](CCCC)(CCCC)CCCC | TFSI | O=S([N-]S(=O)(C(F)(F)F)=O)(C(F)(F)F)=O |
| **IL275** | N4222 | CCCC[N+](CC)(CC)CC | TFSI | O=S([N-]S(=O)(C(F)(F)F)=O)(C(F)(F)F)=O |
| **IL276** | N112Bn | C[N+](C)(CC1=CC=CC=C1)CC | TFSI | O=S([N-]S(=O)(C(F)(F)F)=O)(C(F)(F)F)=O |
| **IL277** | DEME | C[N+](CC)(CCOC)CC | TFSI | O=S([N-]S(=O)(C(F)(F)F)=O)(C(F)(F)F)=O |
| **IL278** | N2444 | CCCC[N+](CC)(CCCC)CCCC | TFSI | O=S([N-]S(=O)(C(F)(F)F)=O)(C(F)(F)F)=O |
| **IL279** | N22214 | CCCCCCCCCCCCCC[N+](CC)(CC)CC | TFSI | O=S([N-]S(=O)(C(F)(F)F)=O)(C(F)(F)F)=O |
| **IL280** | N2666 | CCCCCC[N+](CCCCCC)(CCCCCC)CC | TFSI | O=S([N-]S(=O)(C(F)(F)F)=O)(C(F)(F)F)=O |
|  |  |  |  |  |

Sheet 2: DFT calculation data

**IL** cation cation cation cation anion anion anion anion

cation name anion name

**number** HOMO LUMO dipole volume HOMO LUMO dipole volume

**IL001** C2mim TFSI -0.43409 -0.18524 1.6686 1175.109 -0.13153 0.16539 4.8584 1159.339

**IL002** C4mim TFSI -0.42888 -0.18343 5.0149 1450.197 -0.13153 0.16539 4.8584 1159.339

**IL003** C6mim NTf2 -0.40716 -0.18081 10.6268 1824.281 -0.13153 0.16539 4.8583 1333.241

**IL004** C8mim TFSI -0.38813 -0.18003 15.1765 2122.767 -0.13153 0.16539 4.8584 1159.339

**IL005** C10mim TFSI -0.36883 -0.17981 20.4968 1783.19 -0.13153 0.16539 4.8584 1159.339

**IL006** C12mim TFSI -0.35229 -0.18015 27.2247 2589.398 -0.13153 0.16539 4.8584 1159.339

**IL007** C4mim NFSI -0.42888 -0.18343 5.0149 1450.197 -0.14467 0.10272 9.2588 2035.11

**IL008** C6mim NFSI -0.40716 -0.18081 10.6268 1824.281 -0.14467 0.10272 9.2588 2035.11

**IL009** C8mim NFSI -0.38813 -0.18003 15.1765 2122.767 -0.14467 0.10272 9.2588 2035.11

**IL010** C4Py TFSI -0.45492 -0.24158 3.8624 1404.726 -0.13153 0.16539 4.8584 1159.339

**IL011** N14Pyrr TFSI -0.45312 -0.11255 3.8995 1669.89 -0.13153 0.16539 4.8584 1159.339

**IL012** N14Pipe TFSI -0.45159 -0.11064 3.278 1773.759 -0.13153 0.16539 4.8584 1159.339

**IL013** C6Py TFSI -0.41138 -0.24042 9.4125 1617.842 -0.13153 0.16539 4.8584 1159.339

**IL014** N13Pipe TFSI -0.47489 -0.11194 2.1079 1502.953 -0.13153 0.16539 4.8584 1159.339

**IL015** C4mim HSO4 -0.42888 -0.18343 5.0149 1450.197 -0.05193 0.22943 2.0565 604.037

**IL016** C3mim BF4 -0.4306 -0.18471 3.1244 1356.843 -0.12703 0.39595 0 435.632

**IL017** C2mim acetate -0.43409 -0.18524 1.6686 1175.109 0.0065 0.24455 2.8798 527.306

**IL018** C4mim acetate -0.42888 -0.18343 5.0149 1450.197 0.0065 0.24455 2.8798 527.306

**IL019** C2mim CF3COO -0.43409 -0.18524 1.6686 1175.109 -0.03873 0.23072 4.5389 610.815

**IL020** C2mim C8-sulfate -0.43409 -0.18524 1.6686 1175.109 -0.07235 0.14026 10.8324 2118.525

**IL021** C4mim DCA -0.42888 -0.18343 5.0149 1450.197 -0.03495 0.24356 0.8882 481.042

**IL022** C4mim TCN -0.42888 -0.18343 5.0149 1450.197 -0.0092 0.27557 1.466 656.907

**IL023** C6mim P3C2F5 -0.40716 -0.18081 10.6268 1824.281 -0.16347 0.14427 2.3401 1780.472

**IL024** C2mim MePho -0.43409 -0.18524 1.6686 1175.109 -0.04397 0.21809 3.3247 693.969

**IL025** C10mim Br -0.36883 -0.17981 20.4968 1783.19 0.02009 0.62178 0 344.243

**IL026** C8mim Cl -0.38813 -0.18003 15.1765 2122.767 0.02748 0.82484 0 212.496

**IL027** C8mim Br -0.38813 -0.18003 15.1765 2122.767 0.02009 0.62178 0 344.243

**IL028** C6mim Br -0.40716 -0.18081 10.6268 1824.281 0.02009 0.62178 0 344.243

**IL029** P2225 TFSI -0.42406 -0.1052 4.9 2101.399 -0.13153 0.16539 4.8584 1159.339

**IL030** P222(C1O) NTf2 -0.41398 -0.1141 1.2476 1528.029 -0.13153 0.16539 4.8583 1333.241

**IL031** P222(C2O) NTf2 -0.38028 -0.10851 3.2334 1949.958 -0.13153 0.16539 4.8583 1333.241

| **IL032** | P2228 | TFSI | -0.39567 | -0.10474 | 8.9796 | 2163.575 | -0.13153 | 0.16539 | 4.8584 | 1159.339 |
| --- | --- | --- | --- | --- | --- | --- | --- | --- | --- | --- |
| **IL033** | P4441 | TFSI | -0.4418 | -0.10264 | 0.8495 | 2191.811 | -0.13153 | 0.16539 | 4.8584 | 1159.339 |
| **IL034** | P66614 | FSI | -0.3413 | -0.0969 | 15.0865 | 5329.047 | -0.13482 | 0.14877 | 0.1834 | 971.191 |
| **IL035** | P66614 | TFSI | -0.3413 | -0.0969 | 15.0865 | 5329.047 | -0.13153 | 0.16539 | 4.8584 | 1159.339 |
| **IL036** | P66614 | CTFSI | -0.3413 | -0.0969 | 15.0865 | 5329.047 | -0.13139 | 0.13368 | 5.1508 | 1129.568 |
| **IL037** | P66614 | BETI | -0.3413 | -0.0969 | 15.0865 | 5329.047 | -0.13903 | 0.13382 | 5.7116 | 1481.961 |
| **IL038** | P66614 | NFSI | -0.3413 | -0.0969 | 15.0865 | 5329.047 | -0.14467 | 0.10272 | 9.2588 | 2035.11 |
| **IL039** | P8888 | NFSI | -0.38112 | -0.09594 | 2.821 | 6628.365 | -0.14467 | 0.10272 | 9.2588 | 2035.11 |
| **IL040** | P8888 | TFSI | -0.38112 | -0.09594 | 2.821 | 6628.365 | -0.13153 | 0.16539 | 4.8584 | 1159.339 |
| **IL041** | P8888 | CTFSI | -0.38112 | -0.09594 | 2.821 | 6628.365 | -0.13139 | 0.13368 | 5.1508 | 1129.568 |
| **IL042** | P8888 | BETI | -0.38112 | -0.09594 | 2.821 | 6628.365 | -0.13903 | 0.13382 | 5.7116 | 1481.961 |
| **IL043** | P66614 | Br | -0.3413 | -0.0969 | 15.0865 | 5329.047 | 0.02009 | 0.62178 | 0 | 344.243 |
| **IL044** | C2mim | BF4 | -0.43409 | -0.18524 | 1.6686 | 1175.109 | -0.12703 | 0.39595 | 0 | 435.632 |
| **IL045** | C4mim | BF4 | -0.42888 | -0.18343 | 5.0149 | 1450.197 | -0.12703 | 0.39595 | 0 | 435.632 |
| **IL046** | C2dmim | BF4 | -0.41544 | -0.17487 | 1.3721 | 1380.225 | -0.12703 | 0.39595 | 0 | 435.632 |
| **IL047** | C2dmim | TFSI | -0.41544 | -0.17487 | 1.3721 | 1380.225 | -0.13153 | 0.16539 | 4.8584 | 1159.339 |
| **IL048** | C8mim | BF4 | -0.38813 | -0.18003 | 15.1765 | 2122.767 | -0.12703 | 0.39595 | 0 | 435.632 |
| **IL049** | C2mimOH | BF4 | -0.37218 | -0.25086 | 5.4877 | 1172.188 | -0.12703 | 0.39595 | 0 | 435.632 |
| **IL050** | C2mimOH | TFSI | -0.37218 | -0.25086 | 5.4877 | 1172.188 | -0.13153 | 0.16539 | 4.8584 | 1159.339 |
| **IL051** | DEME | BF4 | -0.38882 | -0.11256 | 3.5848 | 1358.742 | -0.12703 | 0.39595 | 0 | 435.632 |
| **IL052** | N14Pyrr | BF4 | -0.45312 | -0.11255 | 3.8995 | 1669.89 | -0.12703 | 0.39595 | 0 | 435.632 |
| **IL053** | C2mim | HSO4 | -0.43409 | -0.18524 | 1.6686 | 1175.109 | -0.05193 | 0.22943 | 2.0565 | 604.037 |
| **IL054** | C0mim | TFSI | -0.44976 | -0.20106 | 1.4699 | 675.463 | -0.13153 | 0.16539 | 4.8584 | 1159.339 |
| **IL055** | dema | TfO | -0.52384 | -0.14131 | 0.2918 | 1128.058 | -0.06959 | 0.25712 | 4.0434 | 701.596 |
| **IL056** | C4mim | I | -0.42888 | -0.18343 | 5.0149 | 1450.197 | -0.02193 | 0.36105 | 0 | 494.584 |
| **IL057** | C4mim | PF6 | -0.42888 | -0.18343 | 5.0149 | 1450.197 | -0.16273 | 0.2477 | 0 | 524.728 |
| **IL058** | C6mim | PF6 | -0.40716 | -0.18081 | 10.6268 | 1824.281 | -0.16273 | 0.2477 | 0 | 524.728 |
| **IL059** | C8mim | PF6 | -0.38813 | -0.18003 | 15.1765 | 2122.767 | -0.16273 | 0.2477 | 0 | 524.728 |
| **IL060** | C4Py | BF4 | -0.45492 | -0.24158 | 3.8624 | 1404.726 | -0.12703 | 0.39595 | 0 | 435.632 |
| **IL061** | C3Py | BF4 | -0.46226 | -0.24299 | 1.9552 | 1244.466 | -0.12703 | 0.39595 | 0 | 435.632 |
| **IL062** | 2-MeC4Py | BF4 | -0.44231 | -0.23314 | 3.3417 | 1972.9 | -0.12703 | 0.39595 | 0 | 435.632 |
| **IL063** | 4-MeC4Py | BF4 | -0.44957 | -0.22939 | 3.2184 | 1505.79 | -0.12703 | 0.39595 | 0 | 435.632 |
| **IL064** | 3-MeC8Py | BF4 | -0.39048 | -0.23227 | 12.5302 | 2238.668 | -0.12703 | 0.39595 | 0 | 435.632 |
| **IL065** | C5mim | BF4 | -0.423 | -0.18123 | 8.14 | 1271.148 | -0.12703 | 0.39595 | 0 | 435.632 |

| **IL066** | C4mimCN | BF4 | -0.38743 | -0.26165 | 8.4571 | 1570.605 | -0.12703 | 0.39595 | 0 | 435.632 |
| --- | --- | --- | --- | --- | --- | --- | --- | --- | --- | --- |
| **IL067** | C4dmim | BF4 | -0.41232 | -0.17154 | 4.2424 | 1376.728 | -0.12703 | 0.39595 | 0 | 435.632 |
| **IL068** | C6Py | BF4 | -0.41138 | -0.24042 | 9.4125 | 1617.842 | -0.12703 | 0.39595 | 0 | 435.632 |
| **IL069** | 1-Ph 3,5-Me 2-Pen  Pyrazo | BF4 | -0.32901 | -0.24294 | 5.215 | 2292.039 | -0.12703 | 0.39595 | 0 | 435.632 |
| **IL070** | 1-Ph 3,5-Me 2-Hex  Pyrazo | BF4 | -0.39129 | -0.173 | 4.5858 | 2209.514 | -0.12703 | 0.39595 | 0 | 435.632 |
| **IL071** | C5mim | PF6 | -0.423 | -0.18123 | 8.14 | 1271.148 | -0.16273 | 0.2477 | 0 | 524.728 |
| **IL072** | C9mim | PF6 | -0.3777 | -0.17993 | 17.8649 | 2059.345 | -0.16273 | 0.2477 | 0 | 524.728 |
| **IL073** | C2Py | TFSI | -0.46557 | -0.2455 | 0.5883 | 936.709 | -0.13153 | 0.16539 | 4.8584 | 1159.339 |
| **IL074** | N1113 | TFSI | -0.48608 | -0.13026 | 3.0441 | 1020.39 | -0.13153 | 0.16539 | 4.8584 | 1159.339 |
| **IL075** | N1114 | TFSI | -0.45611 | -0.12878 | 5.1428 | 1449.316 | -0.13153 | 0.16539 | 4.8584 | 1159.339 |
| **IL076** | 2-MeC2Py | TFSI | -0.44467 | -0.23291 | 0.0724 | 1146.667 | -0.13153 | 0.16539 | 4.8584 | 1159.339 |
| **IL077** | Ciso4mim | TFSI | -0.42857 | -0.18332 | 4.5485 | 1228.812 | -0.13153 | 0.16539 | 4.8584 | 1159.339 |
| **IL078** | C3mim | TFSI | -0.4306 | -0.18471 | 3.1244 | 1356.843 | -0.13153 | 0.16539 | 4.8584 | 1159.339 |
| **IL079** | N13Pyrr | TFSI | -0.4809 | -0.11387 | 2.1525 | 1173.283 | -0.13153 | 0.16539 | 4.8584 | 1159.339 |
| **IL080** | N2225 | TFSI | -0.42849 | -0.09747 | 5.0342 | 1942.311 | -0.13153 | 0.16539 | 4.8584 | 1159.339 |
| **IL081** | 3-MeC3Py | TFSI | -0.44224 | -0.23552 | 1.5551 | 1340.443 | -0.13153 | 0.16539 | 4.8584 | 1159.339 |
| **IL082** | 3-MeC4Py | TFSI | -0.44045 | -0.23423 | 3.2337 | 1706.532 | -0.13153 | 0.16539 | 4.8584 | 1159.339 |
| **IL083** | 4-MeC4Py | TFSI | -0.44957 | -0.22939 | 3.2184 | 1505.79 | -0.13153 | 0.16539 | 4.8584 | 1159.339 |
| **IL084** | 2-MeC3Py | TFSI | -0.44414 | -0.23451 | 1.6679 | 1316.291 | -0.13153 | 0.16539 | 4.8584 | 1159.339 |
| **IL085** | NH2-Im-C4-Im-NH2 | TFSI | -0.45138 | -0.24849 | 4.035 | 2257.601 | -0.13153 | 0.16539 | 4.8584 | 1159.339 |
| **IL086** | C10Py | TFSI | -0.36853 | -0.23976 | 20.2679 | 2334.832 | -0.13153 | 0.16539 | 4.8584 | 1159.339 |
| **IL087** | N1iso4Pipe | TFSI | -0.45439 | -0.11021 | 3.2608 | 1495.076 | -0.13153 | 0.16539 | 4.8584 | 1159.339 |
| **IL088** | 2-Pe3,5-MeC2Py | TFSI | -0.40709 | -0.21497 | 5.4506 | 1846.299 | -0.13153 | 0.16539 | 4.8584 | 1159.339 |
| **IL089** | 2-Pe3,5-MeC4Py | TFSI | -0.39818 | -0.21045 | 3.874 | 3051.097 | -0.13153 | 0.16539 | 4.8584 | 1159.339 |
| **IL090** | 2,3,5-MeC6Py | TFSI | -0.41146 | -0.21707 | 6.1512 | 2141.008 | -0.13153 | 0.16539 | 4.8584 | 1159.339 |
| **IL091** | 2-Et3,5-MeC6Py | TFSI | -0.40941 | -0.21319 | 4.6693 | 2205.864 | -0.13153 | 0.16539 | 4.8584 | 1159.339 |
| **IL092** | 2-Pe3,5-MeC6Py | TFSI | -0.39718 | -0.20915 | 5.7956 | 2151.145 | -0.13153 | 0.16539 | 4.8584 | 1159.339 |
| **IL093** | C12Py | TFSI | -0.35411 | -0.23967 | 25.9743 | 2174.259 | -0.13153 | 0.16539 | 4.8584 | 1159.339 |
| **IL094** | C14mim | TFSI | -0.34369 | -0.17969 | 31.6537 | 3078.76 | -0.13153 | 0.16539 | 4.8584 | 1159.339 |
| **IL095** | N1iso4Pyrr | TFSI | -0.46986 | -0.11442 | 2.4249 | 1607.098 | -0.13153 | 0.16539 | 4.8584 | 1159.339 |
| **IL096** | 3-MeCiso4Py | TFSI | -0.44018 | -0.23376 | 2.6544 | 1577 | -0.13153 | 0.16539 | 4.8584 | 1159.339 |
| **IL097** | C44im | TFSI | -0.37243 | -0.24026 | 3.5989 | 1882.82 | -0.13153 | 0.16539 | 4.8584 | 1159.339 |
| **IL098** | 3,5-MeC6Py | TFSI | -0.40104 | -0.27707 | 5.7238 | 1881.754 | -0.13153 | 0.16539 | 4.8584 | 1159.339 |
| **IL099** | N2228 | TFSI | -0.39886 | -0.0975 | 9.1309 | 2089.283 | -0.13153 | 0.16539 | 4.8584 | 1159.339 |

| **IL100** | C3CN2O2im | TFSI | -0.36888 | -0.24988 | 7.7657 | 1605.726 | -0.13153 | 0.16539 | 4.8584 | 1159.339 |
| --- | --- | --- | --- | --- | --- | --- | --- | --- | --- | --- |
| **IL101** | 3-MeC4Py | DCA | -0.44045 | -0.23423 | 3.2337 | 1706.532 | -0.03495 | 0.24356 | 0.8882 | 481.042 |
| **IL102** | P66614 | DCA | -0.3413 | -0.0969 | 15.0865 | 5329.047 | -0.03495 | 0.24356 | 0.8882 | 481.042 |
| **IL103** | 3-MeC2Py | FSI | -0.44531 | -0.23785 | 0.8048 | 1322.819 | -0.13482 | 0.14877 | 0.1834 | 971.191 |
| **IL104** | C6Py | FSI | -0.41138 | -0.24042 | 9.4125 | 1617.842 | -0.13482 | 0.14877 | 0.1834 | 971.191 |
| **IL105** | C4Py | FSI | -0.45492 | -0.24158 | 3.8624 | 1404.726 | -0.13482 | 0.14877 | 0.1834 | 971.191 |
| **IL106** | C2Py | FSI | -0.46557 | -0.2455 | 0.5883 | 936.709 | -0.13482 | 0.14877 | 0.1834 | 971.191 |
| **IL107** | N13Pyrr | FSI | -0.4809 | -0.11387 | 2.1525 | 1173.283 | -0.13482 | 0.14877 | 0.1834 | 971.191 |
| **IL108** | C2mim | FSI | -0.43409 | -0.18524 | 1.6686 | 1175.109 | -0.13482 | 0.14877 | 0.1834 | 971.191 |
| **IL109** | N14Pyrr | FSI | -0.45312 | -0.11255 | 3.8995 | 1669.89 | -0.13482 | 0.14877 | 0.1834 | 971.191 |
| **IL110** | N14Pyrr | DCA | -0.45312 | -0.11255 | 3.8995 | 1669.89 | -0.03495 | 0.24356 | 0.8882 | 481.042 |
| **IL111** | C6mim | DCA | -0.40716 | -0.18081 | 10.6268 | 1824.281 | -0.03495 | 0.24356 | 0.8882 | 481.042 |
| **IL112** | Choline | NFSI | -0.37702 | -0.23791 | 2.2567 | 895.068 | -0.14467 | 0.10272 | 9.2588 | 2035.11 |
| **IL113** | C2mC1O | DCA | -0.36711 | -0.24488 | 5.3512 | 1193.062 | -0.03495 | 0.24356 | 0.8882 | 481.042 |
| **IL114** | C18dienmim | TFSI | -0.2795 | -0.17962 | 37.0112 | 3931.955 | -0.13153 | 0.16539 | 4.8584 | 1159.339 |
| **IL115** | C18monoenmim | TFSI | -0.33367 | -0.24365 | 8.4632 | 3745.584 | -0.13153 | 0.16539 | 4.8584 | 1159.339 |
| **IL116** | C4Py | DCA | -0.45492 | -0.24158 | 3.8624 | 1404.726 | -0.03495 | 0.24356 | 0.8882 | 481.042 |
| **IL117** | C3Py | DCA | -0.46226 | -0.24299 | 1.9552 | 1244.466 | -0.03495 | 0.24356 | 0.8882 | 481.042 |
| **IL118** | C6Py | DCA | -0.41138 | -0.24042 | 9.4125 | 1617.842 | -0.03495 | 0.24356 | 0.8882 | 481.042 |
| **IL119** | C3CN2O2im | DCA | -0.36888 | -0.24988 | 7.7657 | 1605.726 | -0.03495 | 0.24356 | 0.8882 | 481.042 |
| **IL120** | C4CNmim | TFSI | -0.37917 | -0.25148 | 9.0894 | 1369.845 | -0.13153 | 0.16539 | 4.8584 | 1159.339 |
| **IL121** | N1888 | TFSI | -0.38618 | -0.09224 | 1.2999 | 3907.608 | -0.13153 | 0.16539 | 4.8584 | 1159.339 |
| **IL122** | N110 Pyrr | TFSI | -0.38045 | -0.11197 | 14.0193 | 2825.466 | -0.13153 | 0.16539 | 4.8584 | 1159.339 |
| **IL123** | N113iso3 | TFSI | -0.47929 | -0.11228 | 1.5235 | 1239.923 | -0.13153 | 0.16539 | 4.8584 | 1159.339 |
| **IL124** | N12OH Pyrr | TFSI | -0.42664 | -0.11494 | 1.769 | 1166.59 | -0.13153 | 0.16539 | 4.8584 | 1159.339 |
| **IL125** | N116iso3 | TFSI | -0.41255 | -0.10979 | 7.6853 | 1869.387 | -0.13153 | 0.16539 | 4.8584 | 1159.339 |
| **IL126** | N1110iso3 | TFSI | -0.37915 | -0.10885 | 13.8002 | 2286.692 | -0.13153 | 0.16539 | 4.8584 | 1159.339 |
| **IL127** | N112OHiso3 | TFSI | -0.41362 | -0.1169 | 2.4286 | 1368.632 | -0.13153 | 0.16539 | 4.8584 | 1159.339 |
| **IL128** | N1(OH)2 Pyrr | TFSI | -0.40391 | -0.11218 | 3.7885 | 1505.681 | -0.13153 | 0.16539 | 4.8584 | 1159.339 |
| **IL129** | N11114 | TFSI | -0.34689 | -0.12571 | 30.0634 | 2978.761 | -0.13153 | 0.16539 | 4.8584 | 1159.339 |
| **IL130** | N22210 | TFSI | -0.37893 | -0.0972 | 13.0322 | 2890.959 | -0.13153 | 0.16539 | 4.8584 | 1159.339 |
| **IL131** | N22212 | TFSI | -0.35449 | -0.09635 | 22.2719 | 3733.452 | -0.13153 | 0.16539 | 4.8584 | 1159.339 |
| **IL132** | C6Diaza | TFSI | -0.37742 | -0.12245 | 4.8119 | 1877.773 | -0.13153 | 0.16539 | 4.8584 | 1159.339 |
| **IL133** | 3,5-MeC4Py | TFSI | -0.4294 | -0.22739 | 2.2865 | 1535.238 | -0.13153 | 0.16539 | 4.8584 | 1159.339 |

| **IL134** | 2-Me,5-EtC4Py | TFSI | -0.42343 | -0.22354 | 2.6898 | 1630.095 | -0.13153 | 0.16539 | 4.8584 | 1159.339 |
| --- | --- | --- | --- | --- | --- | --- | --- | --- | --- | --- |
| **IL135** | 2,3,5-MeC4Py | TFSI | -0.41413 | -0.21609 | 2.3405 | 1932.655 | -0.13153 | 0.16539 | 4.8584 | 1159.339 |
| **IL136** | 3,5-MeC8Py | TFSI | -0.36573 | -0.28644 | 11.8388 | 2199.594 | -0.13153 | 0.16539 | 4.8584 | 1159.339 |
| **IL137** | 2,3-MeC8Py | TFSI | -0.38662 | -0.22282 | 12.3231 | 2335.48 | -0.13153 | 0.16539 | 4.8584 | 1159.339 |
| **IL138** | 2,3,5-MeC8Py | TFSI | -0.39512 | -0.21596 | 8.9204 | 2296.022 | -0.13153 | 0.16539 | 4.8584 | 1159.339 |
| **IL139** | 2,3-MeC4Py | TFSI | -0.42612 | -0.22507 | 2.7101 | 1352.841 | -0.13153 | 0.16539 | 4.8584 | 1159.339 |
| **IL140** | 2,4-MeC6Py | TFSI | -0.41172 | -0.21985 | 6.9942 | 1698.711 | -0.13153 | 0.16539 | 4.8584 | 1159.339 |
| **IL141** | C4CNdmim | TFSI | -0.41005 | -0.17052 | 3.5561 | 1675.734 | -0.13153 | 0.16539 | 4.8584 | 1159.339 |
| **IL142** | C4CNdmim | DCA | -0.41005 | -0.17052 | 3.5561 | 1675.734 | -0.03495 | 0.24356 | 0.8882 | 481.042 |
| **IL143** | C4PyCN | TFSI | -0.44258 | -0.24957 | 7.5749 | 1471.547 | -0.13153 | 0.16539 | 4.8584 | 1159.339 |
| **IL144** | C4PyCN | DCA | -0.44258 | -0.24957 | 7.5749 | 1471.547 | -0.03495 | 0.24356 | 0.8882 | 481.042 |
| **IL145** | C4mimCN | DCA | -0.38743 | -0.26165 | 8.4571 | 1570.605 | -0.03495 | 0.24356 | 0.8882 | 481.042 |
| **IL146** | C8mim | TCN | -0.38813 | -0.18003 | 15.1765 | 2122.767 | -0.0092 | 0.27557 | 1.466 | 656.907 |
| **IL147** | C6mim | TCN | -0.40716 | -0.18081 | 10.6268 | 1824.281 | -0.0092 | 0.27557 | 1.466 | 656.907 |
| **IL148** | 3,5-MeC8Py | TCN | -0.36573 | -0.28644 | 11.8388 | 2199.594 | -0.0092 | 0.27557 | 1.466 | 656.907 |
| **IL149** | 2,3-MeC8Py | TCN | -0.38662 | -0.22282 | 12.3231 | 2335.48 | -0.0092 | 0.27557 | 1.466 | 656.907 |
| **IL150** | P4442 | diEt-  Phosphate | -0.43867 | -0.10453 | 2.3147 | 2612.351 | -0.05947 | 0.18228 | 5.4254 | 1250.183 |
| **IL151** | C2mim | P3C2F5 | -0.43409 | -0.18524 | 1.6686 | 1175.109 | -0.16347 | 0.14427 | 2.3401 | 1780.472 |
| **IL152** | C2mim | diMe-  Phosphate | -0.43409 | -0.18524 | 1.6686 | 1175.109 | -0.05493 | 0.20622 | 4.1517 | 875.172 |
| **IL153** | C2mim | diEt-  Phosphate | -0.43409 | -0.18524 | 1.6686 | 1175.109 | -0.05947 | 0.18228 | 5.4254 | 1250.183 |
| **IL154** | P4441 | Me-sulfate | -0.4418 | -0.10264 | 0.8495 | 2191.811 | -0.05789 | 0.21537 | 3.2266 | 636.918 |
| **IL155** | C2mim | Et-sulfate | -0.43409 | -0.18524 | 1.6686 | 1175.109 | -0.06044 | 0.1863 | 4.8878 | 813.301 |
| **IL156** | C4mim | C8-sulfate | -0.42888 | -0.18343 | 5.0149 | 1450.197 | -0.07235 | 0.14026 | 10.8324 | 2118.525 |
| **IL157** | C1mim | Me-sulfate | -0.43875 | -0.18913 | 0.7772 | 785.518 | -0.05789 | 0.21537 | 3.2266 | 636.918 |
| **IL158** | C4mim | Me-sulfate | -0.42888 | -0.18343 | 5.0149 | 1450.197 | -0.05789 | 0.21537 | 3.2266 | 636.918 |
| **IL159** | L-Ala | C12-sulfate | -0.45299 | -0.16831 | 7.9593 | 1172.269 | -0.06546 | 0.10168 | 26.7646 | 2752.613 |
| **IL160** | 2-iso4 Pyrr | C12-sulfate | -0.4301 | -0.15703 | 8.4075 | 1633.711 | -0.06546 | 0.10168 | 26.7646 | 2752.613 |
| **IL161** | 1-iso3 3-Me aminium | C12-sulfate | -0.44733 | -0.16261 | 5.9715 | 1276.406 | -0.06546 | 0.10168 | 26.7646 | 2752.613 |
| **IL162** | 2-iso3 Pyrr | C12-sulfate | -0.44156 | -0.15527 | 6.4485 | 1370.936 | -0.06546 | 0.10168 | 26.7646 | 2752.613 |
| **IL163** | 1-iso4 1-Opr aminium | C12-sulfate | -0.43722 | -0.17018 | 9.9761 | 1439.007 | -0.06546 | 0.10168 | 26.7646 | 2752.613 |
| **IL164** | 1,5-iso3 1,5-dioxo5 aminium | C12-sulfate | -0.42774 | -0.14593 | 2.4828 | 1960.226 | -0.06546 | 0.10168 | 26.7646 | 2752.613 |
| **IL165** | 2-EtC1Py | Me-sulfate | -0.44611 | -0.23603 | 2.1003 | 1179.051 | -0.05789 | 0.21537 | 3.2266 | 636.918 |
| **IL166** | 2-EtC2Py | Et-sulfate | -0.43955 | -0.22992 | 1.0996 | 1239.95 | -0.06044 | 0.1863 | 4.8878 | 813.301 |
| **IL167** | C1Py | Me-sulfate | -0.47056 | -0.25076 | 0.991 | 829.62 | -0.05789 | 0.21537 | 3.2266 | 636.918 |

| **IL168** | 3-MeC1Py | Me-sulfate | -0.45009 | -0.24273 | 1.7768 | 1061.204 | -0.05789 | 0.21537 | 3.2266 | 636.918 |
| --- | --- | --- | --- | --- | --- | --- | --- | --- | --- | --- |
| **IL169** | C2Py | Et-sulfate | -0.46557 | -0.2455 | 0.5883 | 936.709 | -0.06044 | 0.1863 | 4.8878 | 813.301 |
| **IL170** | C2mim | Me-sulfate | -0.43409 | -0.18524 | 1.6686 | 1175.109 | -0.05789 | 0.21537 | 3.2266 | 636.918 |
| **IL171** | Cbnmim | Me-sulfate | -0.36265 | -0.24297 | 2.9499 | 1370.438 | -0.05789 | 0.21537 | 3.2266 | 636.918 |
| **IL172** | 3-MeC2Py | Et-sulfate | -0.44531 | -0.23785 | 0.8048 | 1322.819 | -0.06044 | 0.1863 | 4.8878 | 813.301 |
| **IL173** | N14Pyrr | Me-sulfate | -0.45312 | -0.11255 | 3.8995 | 1669.89 | -0.05789 | 0.21537 | 3.2266 | 636.918 |
| **IL174** | N2221 | Me-sulfate | -0.51063 | -0.11578 | 0.8267 | 1100.934 | -0.05789 | 0.21537 | 3.2266 | 636.918 |
| **IL175** | N12Pyrr | Et-sulfate | -0.49847 | -0.11805 | 0.8718 | 855.642 | -0.06044 | 0.1863 | 4.8878 | 813.301 |
| **IL176** | C4pro-CNim | C12-sulfate | -0.38273 | -0.26732 | 9.7391 | 1826.425 | -0.06546 | 0.10168 | 26.7646 | 2752.613 |
| **IL177** | C10pro-CNim | C12-sulfate | -0.37942 | -0.25724 | 10.6923 | 2719.202 | -0.06546 | 0.10168 | 26.7646 | 2752.613 |
| **IL178** | C8pro-CNim | C10-sulfate | -0.38371 | -0.26063 | 6.3725 | 2902.296 | -0.06539 | 0.10988 | 21.5705 | 2299.991 |
| **IL179** | C6pro-CNim | C12-sulfate | -0.3843 | -0.25604 | 6.3741 | 2090.75 | -0.06546 | 0.10168 | 26.7646 | 2752.613 |
| **IL180** | C0mim | HSO4 | -0.44976 | -0.20106 | 1.4699 | 675.463 | -0.05193 | 0.22943 | 2.0565 | 604.037 |
| **IL181** | C0Buim | HSO4 | -0.43935 | -0.19257 | 6.5433 | 1274.586 | -0.05193 | 0.22943 | 2.0565 | 604.037 |
| **IL182** | C2mim | MeOEtOEt-  sulfate | -0.43409 | -0.18524 | 1.6686 | 1175.109 | -0.06872 | 0.12767 | 14.3329 | 1366.582 |
| **IL183** | N24Pyrr | Et-sulfate | -0.45115 | -0.10535 | 3.2864 | 1708.363 | -0.06044 | 0.1863 | 4.8878 | 813.301 |
| **IL184** | C3mim | Me-sulfate | -0.4306 | -0.18471 | 3.1244 | 1356.843 | -0.05789 | 0.21537 | 3.2266 | 636.918 |
| **IL185** | C6mim | Et-sulfate | -0.40716 | -0.18081 | 10.6268 | 1824.281 | -0.06044 | 0.1863 | 4.8878 | 813.301 |
| **IL186** | C8mim | MeOEtOEt-  sulfate | -0.38813 | -0.18003 | 15.1765 | 2122.767 | -0.06872 | 0.12767 | 14.3329 | 1366.582 |
| **IL187** | N1(EtOH)3 | Me-sulfate | -0.40513 | -0.11039 | 2.6414 | 1394.203 | -0.05789 | 0.21537 | 3.2266 | 636.918 |
| **IL188** | C2etim | Et-sulfate | -0.37908 | -0.25239 | 3.1048 | 1089.826 | -0.06044 | 0.1863 | 4.8878 | 813.301 |
| **IL189** | N0002 | HSO4 | -0.55024 | -0.18859 | 3.9594 | 587.569 | -0.05193 | 0.22943 | 2.0565 | 604.037 |
| **IL190** | C4mim | MeOEtOEt-  sulfate | -0.42888 | -0.18343 | 5.0149 | 1450.197 | -0.06872 | 0.12767 | 14.3329 | 1366.582 |
| **IL191** | N1122OH | Et-sulfate | -0.42899 | -0.11997 | 2.2641 | 1187.893 | -0.06044 | 0.1863 | 4.8878 | 813.301 |
| **IL192** | N1124 | Et-sulfate | -0.45433 | -0.11875 | 4.5086 | 1480.193 | -0.06044 | 0.1863 | 4.8878 | 813.301 |
| **IL193** | N0022 | HSO4 | -0.53263 | -0.15939 | 0.5336 | 756.895 | -0.05193 | 0.22943 | 2.0565 | 604.037 |
| **IL194** | N0111 | HSO4 | -0.5876 | -0.16407 | 0.8916 | 708.041 | -0.05193 | 0.22943 | 2.0565 | 604.037 |
| **IL195** | N0222 | HSO4 | -0.51366 | -0.13697 | 1.0881 | 1223.463 | -0.05193 | 0.22943 | 2.0565 | 604.037 |
| **IL196** | C2Py | TfO | -0.46557 | -0.2455 | 0.5883 | 936.709 | -0.06959 | 0.25712 | 4.0434 | 701.596 |
| **IL197** | C4Py | TfO | -0.45492 | -0.24158 | 3.8624 | 1404.726 | -0.06959 | 0.25712 | 4.0434 | 701.596 |
| **IL198** | C2mim | TfO | -0.43409 | -0.18524 | 1.6686 | 1175.109 | -0.06959 | 0.25712 | 4.0434 | 701.596 |
| **IL199** | C4mim | TfO | -0.42888 | -0.18343 | 5.0149 | 1450.197 | -0.06959 | 0.25712 | 4.0434 | 701.596 |
| **IL200** | N14Pyrr | TfO | -0.45312 | -0.11255 | 3.8995 | 1669.89 | -0.06959 | 0.25712 | 4.0434 | 701.596 |
| **IL201** | 3-MeC4Py | TfO | -0.44045 | -0.23423 | 3.2337 | 1706.532 | -0.06959 | 0.25712 | 4.0434 | 701.596 |

| **IL202** | C42im | TfO | -0.37355 | -0.24747 | 5.1154 | 1342.929 | -0.06959 | 0.25712 | 4.0434 | 701.596 |
| --- | --- | --- | --- | --- | --- | --- | --- | --- | --- | --- |
| **IL203** | 2,3-MeC4Py | TfO | -0.42612 | -0.22507 | 2.7101 | 1352.841 | -0.06959 | 0.25712 | 4.0434 | 701.596 |
| **IL204** | C6mim | TfO | -0.40716 | -0.18081 | 10.6268 | 1824.281 | -0.06959 | 0.25712 | 4.0434 | 701.596 |
| **IL205** | C43CNim | TfO | -0.37679 | -0.25409 | 6.6158 | 1817.864 | -0.06959 | 0.25712 | 4.0434 | 701.596 |
| **IL206** | C63CNim | TfO | -0.37406 | -0.25402 | 5.2977 | 2077.567 | -0.06959 | 0.25712 | 4.0434 | 701.596 |
| **IL207** | C83CNim | TfO | -0.37531 | -0.25216 | 13.9987 | 2173.325 | -0.06959 | 0.25712 | 4.0434 | 701.596 |
| **IL208** | 2,3-MeC8Py | TfO | -0.38662 | -0.22282 | 12.3231 | 2335.48 | -0.06959 | 0.25712 | 4.0434 | 701.596 |
| **IL209** | P66614 | TfO | -0.3413 | -0.0969 | 15.0865 | 5329.047 | -0.06959 | 0.25712 | 4.0434 | 701.596 |
| **IL210** | C4mim | Br | -0.42888 | -0.18343 | 5.0149 | 1450.197 | 0.02009 | 0.62178 | 0 | 344.243 |
| **IL211** | C43CNim | Br | -0.37679 | -0.25409 | 6.6158 | 1817.864 | 0.02009 | 0.62178 | 0 | 344.243 |
| **IL212** | C63CNim | Br | -0.37406 | -0.25402 | 5.2977 | 2077.567 | 0.02009 | 0.62178 | 0 | 344.243 |
| **IL213** | C83CNim | Br | -0.37531 | -0.25216 | 13.9987 | 2173.325 | 0.02009 | 0.62178 | 0 | 344.243 |
| **IL214** | P8888 | Br | -0.38112 | -0.09594 | 2.821 | 6628.365 | 0.02009 | 0.62178 | 0 | 344.243 |
| **IL215** | C3all3CN | Cl | -0.37625 | -0.25631 | 3.7604 | 1520.659 | 0.02748 | 0.82484 | 0 | 212.496 |
| **IL216** | C2OH3CN | Cl | -0.37273 | -0.25212 | 3.7891 | 1708.057 | 0.02748 | 0.82484 | 0 | 212.496 |
| **IL217** | C6mim | Cl | -0.40716 | -0.18081 | 10.6268 | 1824.281 | 0.02748 | 0.82484 | 0 | 212.496 |
| **IL218** | C6OH0im | Cl | -0.34772 | -0.25166 | 10.3796 | 1343.564 | 0.02748 | 0.82484 | 0 | 212.496 |
| **IL219** | CBnmim | Cl | -0.37692 | -0.17377 | 6.2324 | 1671.611 | 0.02748 | 0.82484 | 0 | 212.496 |
| **IL220** | C43CNim | Cl | -0.37679 | -0.25409 | 6.6158 | 1817.864 | 0.02748 | 0.82484 | 0 | 212.496 |
| **IL221** | C6OHmim | Cl | -0.34651 | -0.25049 | 8.871 | 1607.809 | 0.02748 | 0.82484 | 0 | 212.496 |
| **IL222** | C63CNim | Cl | -0.37406 | -0.25402 | 5.2977 | 2077.567 | 0.02748 | 0.82484 | 0 | 212.496 |
| **IL223** | C10mim | Cl | -0.36883 | -0.17981 | 20.4968 | 1783.19 | 0.02748 | 0.82484 | 0 | 212.496 |
| **IL224** | C46OHim | Cl | -0.37759 | -0.16505 | 3.3781 | 2380.388 | 0.02748 | 0.82484 | 0 | 212.496 |
| **IL225** | C83CNim | Cl | -0.37531 | -0.25216 | 13.9987 | 2173.325 | 0.02748 | 0.82484 | 0 | 212.496 |
| **IL226** | P4448 | Cl | -0.38349 | -0.1001 | 6.944 | 3629.171 | 0.02748 | 0.82484 | 0 | 212.496 |
| **IL227** | P66614 | Cl | -0.3413 | -0.0969 | 15.0865 | 5329.047 | 0.02748 | 0.82484 | 0 | 212.496 |
| **IL228** | N0002 | acetate | -0.55024 | -0.18859 | 3.9594 | 587.569 | 0.0065 | 0.24455 | 2.8798 | 527.306 |
| **IL229** | N0111 | acetate | -0.5876 | -0.16407 | 0.8916 | 708.041 | 0.0065 | 0.24455 | 2.8798 | 527.306 |
| **IL230** | N0003 | acetate | -0.50176 | -0.18522 | 6.4429 | 704.385 | 0.0065 | 0.24455 | 2.8798 | 527.306 |
| **IL231** | N0002 | glycolate | -0.55024 | -0.18859 | 3.9594 | 587.569 | -0.02056 | 0.24946 | 2.2619 | 626.26 |
| **IL232** | N0004 | acetate | -0.4665 | -0.18328 | 8.9365 | 747.334 | 0.0065 | 0.24455 | 2.8798 | 527.306 |
| **IL233** | N0022 | acetate | -0.53263 | -0.15939 | 0.5336 | 756.895 | 0.0065 | 0.24455 | 2.8798 | 527.306 |
| **IL234** | N0112 | acetate | -0.53271 | -0.15215 | 1.589 | 874.459 | 0.0065 | 0.24455 | 2.8798 | 527.306 |
| **IL235** | N0012OH | acetate | -0.45492 | -0.15461 | 2.2824 | 684.878 | 0.0065 | 0.24455 | 2.8798 | 527.306 |

| **IL236** | C0mim | acetate | -0.44976 | -0.20106 | 1.4699 | 675.463 | 0.0065 | 0.24455 | 2.8798 | 527.306 |
| --- | --- | --- | --- | --- | --- | --- | --- | --- | --- | --- |
| **IL237** | N0222 | acetate | -0.51366 | -0.13697 | 1.0881 | 1223.463 | 0.0065 | 0.24455 | 2.8798 | 527.306 |
| **IL238** | N1112OH | glycinate | -0.43587 | -0.12749 | 1.6939 | 1053.964 | -0.0045 | 0.22723 | 3.1565 | 674.8 |
| **IL239** | C03CNim | acetate | -0.45169 | -0.21295 | 9.8827 | 1108.57 | 0.0065 | 0.24455 | 2.8798 | 527.306 |
| **IL240** | C3mim | acetate | -0.4306 | -0.18471 | 3.1244 | 1356.843 | 0.0065 | 0.24455 | 2.8798 | 527.306 |
| **IL241** | C2mim | ammonioaceta  te | -0.43409 | -0.18524 | 1.6686 | 1175.109 | -0.0045 | 0.22721 | 3.1567 | 698.069 |
| **IL242** | N14Pyrr | acetate | -0.45312 | -0.11255 | 3.8995 | 1669.89 | 0.0065 | 0.24455 | 2.8798 | 527.306 |
| **IL243** | C5mim | acetate | -0.423 | -0.18123 | 8.14 | 1271.148 | 0.0065 | 0.24455 | 2.8798 | 527.306 |
| **IL244** | C6mim | acetate | -0.40716 | -0.18081 | 10.6268 | 1824.281 | 0.0065 | 0.24455 | 2.8798 | 527.306 |
| **IL245** | CEtOEt3CN | CF3COO | -0.36888 | -0.24988 | 7.7652 | 1918.027 | -0.03873 | 0.23072 | 4.5389 | 610.815 |
| **IL246** | C4mim | ibuprofenate | -0.42888 | -0.18343 | 5.0149 | 1450.197 | -0.03143 | 0.11243 | 14.7852 | 2334.57 |
| **IL247** | P66614 | acetate | -0.3413 | -0.0969 | 15.0865 | 5329.047 | 0.0065 | 0.24455 | 2.8798 | 527.306 |
| **IL248** | N0002 | NO3 | -0.55024 | -0.18859 | 3.9594 | 587.569 | 0.00582 | 0.2225 | 0 | 331.246 |
| **IL249** | N0002OH | NO3 | -0.46344 | -0.17274 | 3.557 | 679.578 | 0.00582 | 0.2225 | 0 | 331.246 |
| **IL250** | Pyrr | NO3 | -0.51674 | -0.16809 | 3.3459 | 609.191 | 0.00582 | 0.2225 | 0 | 331.246 |
| **IL251** | N0004 | NO3 | -0.4665 | -0.18328 | 8.9365 | 747.334 | 0.00582 | 0.2225 | 0 | 331.246 |
| **IL252** | C2mim | Me-sulfonate | -0.43409 | -0.18524 | 1.6686 | 1175.109 | -0.03547 | 0.2223 | 3.3313 | 651.26 |
| **IL253** | C2mim | taurinate | -0.43409 | -0.18524 | 1.6686 | 1175.109 | -0.04375 | 0.16709 | 6.303 | 796.565 |
| **IL254** | N1122OH | Bu-sulfonate | -0.42899 | -0.11997 | 2.2641 | 1187.893 | -0.04365 | 0.16121 | 8.3447 | 950.924 |
| **IL255** | C2mim | 4-Me Bnsulfonate | -0.43409 | -0.18524 | 1.6686 | 1175.109 | -0.05697 | 0.11146 | 9.7364 | 1428.263 |
| **IL256** | 1-Ph 2,3,5-Me Pyraz | Me-sulfonate | -0.35692 | -0.22693 | 5.3818 | 2014.164 | -0.03547 | 0.2223 | 3.3313 | 651.26 |
| **IL257** | C03CNim | 4-Me Bnsulfonate | -0.45169 | -0.21295 | 9.8827 | 1108.57 | -0.05697 | 0.11146 | 9.7364 | 1428.263 |
| **IL258** | N1112OH | OHAmEt-  solfonate | -0.43587 | -0.12749 | 1.6939 | 1053.964 | -0.0582 | 0.14267 | 12.2295 | 1440.708 |
| **IL259** | C2mim | AAMP-  sulfonate | -0.43409 | -0.18524 | 1.6686 | 1175.109 | -0.07265 | 0.05507 | 8.9361 | 1781.681 |
| **IL260** | 1-Ph 2-Bu 3,5-Me  Pyraz | Me-sulfonate | -0.39312 | -0.17324 | 2.6082 | 2587.445 | -0.03547 | 0.2223 | 3.3313 | 651.26 |
| **IL261** | N1112OH | MorOH-  sulfonate | -0.43587 | -0.12749 | 1.6939 | 1053.964 | -0.07429 | 0.14819 | 12.2733 | 1989.392 |
| **IL262** | C2mim | BETI | -0.43409 | -0.18524 | 1.6686 | 1175.109 | -0.13903 | 0.13382 | 5.7116 | 1481.961 |
| **IL263** | C2mim | TCN | -0.43409 | -0.18524 | 1.6686 | 1175.109 | -0.0092 | 0.27557 | 1.466 | 656.907 |
| **IL264** | C2mim | DCA | -0.43409 | -0.18524 | 1.6686 | 1175.109 | -0.03495 | 0.24356 | 0.8882 | 481.042 |
| **IL265** | N1112OH | lactate | -0.43587 | -0.12749 | 1.6939 | 1053.964 | -0.02577 | 0.21891 | 3.3093 | 974.781 |
| **IL266** | C2mim | lactate | -0.43409 | -0.18524 | 1.6686 | 1175.109 | -0.02577 | 0.21891 | 3.3093 | 974.781 |
| **IL267** | S122 | TFSI | -0.49417 | -0.16743 | 1.4675 | 1164.208 | -0.13153 | 0.16539 | 4.8584 | 1159.339 |
| **IL268** | N2OH | HCOO | -0.4634 | -0.17275 | 3.5564 | 460.351 | 0.00971 | 0.30053 | 0.6093 | 369.887 |
| **IL269** | Bmim | BF4 | -0.42616 | -0.17798 | 3.3191 | 1430.735 | -0.12703 | 0.39595 | 0 | 435.632 |

| **IL270** | C6mim | BF4 |  | -0.40716 | -0.18081 | 10.6268 | 1824.281 | -0.12703 | 0.39595 | 0 | 435.632 |
| --- | --- | --- | --- | --- | --- | --- | --- | --- | --- | --- | --- |
| **IL271** | C10mim | BF4 |  | -0.36883 | -0.17981 | 20.4968 | 1783.19 | -0.12703 | 0.39595 | 0 | 435.632 |
| **IL272** | Choline | TFSI |  | -0.37702 | -0.23791 | 2.2567 | 895.068 | -0.13153 | 0.16539 | 4.8584 | 1159.339 |
| **IL273** | N1116 | TFSI |  | -0.42016 | -0.12673 | 9.0116 | 1236.378 | -0.13153 | 0.16539 | 4.8584 | 1159.339 |
| **IL274** | N4441 | TFSI |  | -0.44769 | -0.09686 | 0.5086 | 2092.099 | -0.13153 | 0.16539 | 4.8584 | 1159.339 |
| **IL275** | N4222 | TFSI |  | -0.45002 | -0.09813 | 3.0703 | 1791.728 | -0.13153 | 0.16539 | 4.8584 | 1159.339 |
| **IL276** | N112Bn | TFSI |  | -0.38269 | -0.15603 | 5.2114 | 1568.862 | -0.13153 | 0.16539 | 4.8584 | 1159.339 |
| **IL277** | DEME | TFSI |  | -0.38882 | -0.11256 | 3.5848 | 1358.742 | -0.13153 | 0.16539 | 4.8584 | 1159.339 |
| **IL278** | N2444 | TFSI |  | -0.44579 | -0.09572 | 1.0607 | 1858.961 | -0.13153 | 0.16539 | 4.8584 | 1159.339 |
| **IL279** | N22214 | TFSI |  | -0.35075 | -0.09581 | 22.4449 | 3095.125 | -0.13153 | 0.16539 | 4.8584 | 1159.339 |
| **IL280** | N2666 | TFSI |  | -0.4088 | -0.08555 | 5.3151 | 3291.199 | -0.13153 | 0.16539 | 4.8584 | 1159.339 |
|  |  |  |  |  |  |  |  |  |  |  |  |

Sheet 3: Viability test

**IL number** cation name anion name viability 1H (av.) viability 1H (s.d.) viability 24H (av.) viability 24H (s.d.)

| **IL001** | C2mim | TFSI |  | 0.018822101 |  | 0.018553227 |  | 0.021838253 |  | 0.021761332 |
| --- | --- | --- | --- | --- | --- | --- | --- | --- | --- | --- |
| **IL002** | C4mim | TFSI |  | 0.728293868 |  | 0.050413843 |  | 0.035277178 |  | 0.014165686 |
| **IL003** | C6mim | NTf2 |  | 0.85154827 |  | 0.04411349 |  | 0.051835853 |  | 0.013638182 |
| **IL004** | C8mim | TFSI |  | 0.752580449 |  | 0.126734193 |  | 0.028557715 |  | 0.016336734 |
| **IL005** | C10mim | TFSI |  | 0.032179721 |  | 0.032785498 |  | 0.022798176 |  | 0.011145502 |
| **IL006** | C12mim | TFSI |  | 0.027018822 |  | 0.027402352 |  | 0.033597312 |  | 0.031857321 |
| **IL007** | C4mim | NFSI |  |  |  |  |  |  |  |  |
| **IL008** | C6mim | NFSI |  |  |  |  |  |  |  |  |
| **IL009** | C8mim | NFSI |  |  |  |  |  |  |  |  |
| **IL010** | C4Py | TFSI |  |  |  |  |  |  |  |  |
| **IL011** | N14Pyrr | TFSI |  | 0.875227687 |  | 0.091422098 |  | 0.04799616 |  | 0.010077478 |
| **IL012** | N14Pipe | TFSI |  | 0.887370978 |  | 0.120638118 |  | 0.113510919 |  | 0.02845269 |
| **IL013** | C6Py | TFSI |  |  |  |  |  |  |  |  |
| **IL014** | N13Pipe | TFSI |  |  |  |  |  |  |  |  |
| **IL015** | C4mim | HSO4 |  | 0.009714633 |  | 0.015084945 |  | 0.013918886 |  | 0.014055446 |
| **IL016** | C3mim | BF4 |  | 0.197328476 |  | 0.019014614 |  | 0.031437485 |  | 0.027390185 |
| **IL017** | C2mim | acetate |  | 0.083485124 |  | 0.056758749 |  | 0.026397888 |  | 0.025927249 |
| **IL018** | C4mim | acetate |  | 0.305707347 |  | 0.070768794 |  | 0.019438445 |  | 0.019200694 |
| **IL019** | C2mim | CF3COO |  | 0.418032787 |  | 0.126129563 |  | 0.026157907 |  | 0.023098822 |
| **IL020** | C2mim | C8-sulfate |  |  |  |  |  |  |  |  |
| **IL021** | C4mim | DCA |  |  |  |  |  |  |  |  |
| **IL022** | C4mim | TCN |  | 0.024286582 |  | 0.024689179 |  | 0.027117831 |  | 0.023697163 |
| **IL023** | C6mim | P3C2F5 |  |  |  |  |  |  |  |  |
| **IL024** | C2mim | MePho |  | 0.358834244 |  | 0.164663293 |  | 0.013438925 |  | 0.014290634 |
| **IL025** | C10mim | Br |  | 0.026108075 |  | 0.015151091 |  | 0.023278138 |  | 0.025381533 |
| **IL026** | C8mim | Cl |  | 0.01608986 |  | 0.02498444 |  | 0.023278138 |  | 0.038870154 |
| **IL027** | C8mim | Br |  | 0.013661202 |  | 0.015297059 |  | 0.013678906 |  | 0.013735599 |
| **IL028** | C6mim | Br |  | 0.015482696 |  | 0.024041631 |  | 0.013438925 |  | 0.016213849 |
| **IL029** | P2225 | TFSI |  | 0.78749241 |  | 0.121205977 |  | 0.824094072 |  | 0.037774183 |
| **IL030** | P222(C1O) | NTf2 |  | 0.800546448 |  | 0.036083237 |  | 0.021118311 |  | 0.004988877 |
| **IL031** | P222(C2O) | NTf2 |  | 0.862477231 |  | 0.048627838 |  | 0.072234221 |  | 0.049304045 |

| **IL032** | P2228 | TFSI |  | 1.02003643 |  | 0.158444522 |  | 0.782097432 |  | 0.057389507 |
| --- | --- | --- | --- | --- | --- | --- | --- | --- | --- | --- |
| **IL033** | P4441 | TFSI |  | 0.838797814 |  | 0.209661632 |  | 0.946484281 |  | 0.129074483 |
| **IL034** | P66614 | FSI |  | 0.49058895 |  | 0.20730868 |  | 0.882649388 |  | 0.063644848 |
| **IL035** | P66614 | TFSI |  | 0.648755313 |  | 0.079331933 |  | 1.088552916 |  | 0.005715476 |
| **IL036** | P66614 | CTFSI |  | 0.642683667 |  | 0.175302279 |  | 0.794336453 |  | 0.270688423 |
| **IL037** | P66614 | BETI |  | 0.649058895 |  | 0.093189174 |  | 1.031197504 |  | 0.061081003 |
| **IL038** | P66614 | NFSI |  | 0.70582878 |  | 0.093105675 |  | 1.082073434 |  | 0.018832596 |
| **IL039** | P8888 | NFSI |  | 0.813600486 |  | 0.110530036 |  | 0.999280058 |  | 0.078362406 |
| **IL040** | P8888 | TFSI |  | 0.767152398 |  | 0.100949933 |  | 0.937844972 |  | 0.062237895 |
| **IL041** | P8888 | CTFSI |  | 0.67243473 |  | 0.20015716 |  | 0.707703384 |  | 0.284275688 |
| **IL042** | P8888 | BETI |  | 0.774438373 |  | 0.186075851 |  | 0.723542117 |  | 0.07707572 |
| **IL043** | P66614 | Br |  | 0 |  | 0.014055446 |  | 0 |  | 0.013912425 |
| **IL044** | C2mim | BF4 |  | 0.295385549 |  | 0.023809429 |  | 0.001439885 |  | 0.002828427 |
| **IL045** | C4mim | BF4 |  |  |  |  |  |  |  |  |
| **IL046** | C2dmim | BF4 |  |  |  |  |  |  |  |  |
| **IL047** | C2dmim | TFSI |  | 0.29811779 |  | 0.048030083 |  | 0.011279098 |  | 0.003681787 |
| **IL048** | C8mim | BF4 |  | 0.00941105 |  | 0.01461354 |  | 0 |  | 0.008286535 |
| **IL049** | C2mimOH | BF4 |  | 0.525804493 |  | 0.11870505 |  | 0.002399808 |  | 0.004714045 |
| **IL050** | C2mimOH | TFSI |  | 0.013661202 |  | 0.021213203 |  | 0.005759539 |  | 0.011313708 |
| **IL051** | DEME | BF4 |  | 0.568913175 |  | 0.014007934 |  | 0.002399808 |  | 0.002494438 |
| **IL052** | N14Pyrr | BF4 |  |  |  |  |  |  |  |  |
| **IL053** | C2mim | HSO4 |  |  |  |  |  |  |  |  |
| **IL054** | C0mim | TFSI |  |  |  |  |  |  |  |  |
| **IL055** | dema | TfO |  | 0.007589557 |  | 0.011785113 |  | 0.000719942 |  | 0.001414214 |
| **IL056** | C4mim | I |  |  |  |  |  |  |  |  |
| **IL057** | C4mim | PF6 |  |  |  |  |  |  |  |  |
| **IL058** | C6mim | PF6 |  |  |  |  |  |  |  |  |
| **IL059** | C8mim | PF6 |  |  |  |  |  |  |  |  |
| **IL060** | C4Py | BF4 |  |  |  |  |  |  |  |  |
| **IL061** | C3Py | BF4 |  |  |  |  |  |  |  |  |
| **IL062** | 2-MeC4Py | BF4 |  |  |  |  |  |  |  |  |
| **IL063** | 4-MeC4Py | BF4 |  |  |  |  |  |  |  |  |
| **IL064** | 3-MeC8Py | BF4 |  |  |  |  |  |  |  |  |
| **IL065** | C5mim | BF4 |  |  |  |  |  |  |  |  |

**IL066** C4mimCN BF4

**IL067** C4dmim BF4

**IL068** C6Py BF4

**IL069** 1-Ph 3,5-Me 2-Pen Pyrazo BF4

**IL070** 1-Ph 3,5-Me 2-Hex Pyrazo BF4

**IL071** C5mim PF6

**IL072** C9mim PF6

**IL073** C2Py TFSI

**IL074** N1113 TFSI

**IL075** N1114 TFSI 0.424972618 0.081229442 0.039936314 0.019871811

**IL076** 2-MeC2Py TFSI **IL077** Ciso4mim TFSI

**IL078** C3mim TFSI

**IL079** N13Pyrr TFSI

**IL080** N2225 TFSI 0.86962476 0.123828735 0.401754386 0.060865973

**IL081** 3-MeC3Py TFSI

**IL082** 3-MeC4Py TFSI

**IL083** 4-MeC4Py TFSI

**IL084** 2-MeC3Py TFSI

**IL085** NH2-Im-C4-Im-NH2 TFSI

**IL086** C10Py TFSI

**IL087** N1iso4Pipe TFSI

**IL088** 2-Pe3,5-MeC2Py TFSI

**IL089** 2-Pe3,5-MeC4Py TFSI

**IL090** 2,3,5-MeC6Py TFSI

**IL091** 2-Et3,5-MeC6Py TFSI

**IL092** 2-Pe3,5-MeC6Py TFSI

**IL093** C12Py TFSI

**IL094** C14mim TFSI

**IL095** N1iso4Pyrr TFSI

**IL096** 3-MeCiso4Py TFSI

**IL097** C44im TFSI

**IL098** 3,5-MeC6Py TFSI

**IL099** N2228 TFSI 1.074260679 0.057139206 0.240811994 0.176217668 **IL100** C3CN2O2im TFSI

**IL101** 3-MeC4Py DCA

**IL102** P66614 DCA

**IL103** 3-MeC2Py FSI

**IL104** C6Py FSI

**IL105** C4Py FSI

**IL106** C2Py FSI

**IL107** N13Pyrr FSI

**IL108** C2mim FSI

**IL109** N14Pyrr FSI 0.307586962 0.028110892 0.08625731 0.012119773

**IL110** N14Pyrr DCA

**IL111** C6mim DCA

**IL112** Choline NFSI

**IL113** C2mC1O DCA

**IL114** C18dienmim TFSI

**IL115** C18monoenmim TFSI

**IL116** C4Py DCA

**IL117** C3Py DCA

**IL118** C6Py DCA

**IL119** C3CN2O2im DCA

**IL120** C4CNmim TFSI

**IL121** N1888 TFSI 0.939742536 0.158938842 0.597660819 0.234524104

**IL122** N110 Pyrr TFSI

**IL123** N113iso3 TFSI

**IL124** N12OH Pyrr TFSI

**IL125** N116iso3 TFSI

**IL126** N1110iso3 TFSI

**IL127** N112OHiso3 TFSI

**IL128** N1(OH)2 Pyrr TFSI

**IL129** N11114 TFSI

**IL130** N22210 TFSI

**IL131** N22212 TFSI 0.009725906 0.030165733 0.007430692 0.002442366

**IL132** C6Diaza TFSI

**IL133** 3,5-MeC4Py TFSI

**IL134** 2-Me,5-EtC4Py TFSI

**IL135** 2,3,5-MeC4Py TFSI

**IL136** 3,5-MeC8Py TFSI

**IL137** 2,3-MeC8Py TFSI

**IL138** 2,3,5-MeC8Py TFSI

**IL139** 2,3-MeC4Py TFSI

**IL140** 2,4-MeC6Py TFSI

**IL141** C4CNdmim TFSI

**IL142** C4CNdmim DCA

**IL143** C4PyCN TFSI

**IL144** C4PyCN DCA

**IL145** C4mimCN DCA

**IL146** C8mim TCN

**IL147** C6mim TCN

**IL148** 3,5-MeC8Py TCN

**IL149** 2,3-MeC8Py TCN

**IL150** P4442 diEt-Phosphate

**IL151** C2mim P3C2F5

**IL152** C2mim diMe-Phosphate

**IL153** C2mim diEt-Phosphate

**IL154** P4441 Me-sulfate

**IL155** C2mim Et-sulfate

**IL156** C4mim C8-sulfate

**IL157** C1mim Me-sulfate

**IL158** C4mim Me-sulfate

**IL159** L-Ala C12-sulfate

**IL160** 2-iso4 Pyrr C12-sulfate

**IL161** 1-iso3 3-Me aminium C12-sulfate

**IL162** 2-iso3 Pyrr C12-sulfate

**IL163** 1-iso4 1-Opr aminium C12-sulfate

**IL164** 1,5-iso3 1,5-dioxo5 aminium C12-sulfate

**IL165** 2-EtC1Py Me-sulfate

**IL166** 2-EtC2Py Et-sulfate

**IL167** C1Py Me-sulfate

**IL168** 3-MeC1Py Me-sulfate

**IL169** C2Py Et-sulfate

**IL170** C2mim Me-sulfate

**IL171** Cbnmim Me-sulfate

**IL172** 3-MeC2Py Et-sulfate

**IL173** N14Pyrr Me-sulfate

**IL174** N2221 Me-sulfate

**IL175** N12Pyrr Et-sulfate

**IL176** C4pro-CNim C12-sulfate

**IL177** C10pro-CNim C12-sulfate

**IL178** C8pro-CNim C10-sulfate

**IL179** C6pro-CNim C12-sulfate

**IL180** C0mim HSO4

**IL181** C0Buim HSO4

**IL182** C2mim MeOEtOEt-sulfate

**IL183** N24Pyrr Et-sulfate

**IL184** C3mim Me-sulfate

**IL185** C6mim Et-sulfate

**IL186** C8mim MeOEtOEt-sulfate

**IL187** N1(EtOH)3 Me-sulfate

**IL188** C2etim Et-sulfate

**IL189** N0002 HSO4

**IL190** C4mim MeOEtOEt-sulfate

**IL191** N1122OH Et-sulfate

**IL192** N1124 Et-sulfate

**IL193** N0022 HSO4

**IL194** N0111 HSO4

**IL195** N0222 HSO4

**IL196** C2Py TfO

**IL197** C4Py TfO

**IL198** C2mim TfO

**IL199** C4mim TfO

**IL200** N14Pyrr TfO

**IL201** 3-MeC4Py TfO

**IL202** C42im TfO

**IL203** 2,3-MeC4Py TfO

**IL204** C6mim TfO

**IL205** C43CNim TfO

**IL206** C63CNim TfO

**IL207** C83CNim TfO

**IL208** 2,3-MeC8Py TfO

**IL209** P66614 TfO

**IL210** C4mim Br

**IL211** C43CNim Br

**IL212** C63CNim Br

**IL213** C83CNim Br

**IL214** P8888 Br

**IL215** C3all3CN Cl

**IL216** C2OH3CN Cl

**IL217** C6mim Cl

**IL218** C6OH0im Cl

**IL219** CBnmim Cl

**IL220** C43CNim Cl

**IL221** C6OHmim Cl

**IL222** C63CNim Cl

**IL223** C10mim Cl

**IL224** C46OHim Cl

**IL225** C83CNim Cl

**IL226** P4448 Cl

**IL227** P66614 Cl

**IL228** N0002 acetate

**IL229** N0111 acetate

**IL230** N0003 acetate

**IL231** N0002 glycolate

**IL232** N0004 acetate

**IL233** N0022 acetate

**IL234** N0112 acetate

**IL235** N0012OH acetate

**IL236** C0mim acetate **IL237** N0222 acetate

**IL238** N1112OH glycinate

**IL239** C03CNim acetate

**IL240** C3mim acetate

**IL241** C2mim ammonioacetate

**IL242** N14Pyrr acetate

**IL243** C5mim acetate

**IL244** C6mim acetate

**IL245** CEtOEt3CN CF3COO

**IL246** C4mim ibuprofenate

**IL247** P66614 acetate

**IL248** N0002 NO3

**IL249** N0002OH NO3

**IL250** Pyrr NO3

**IL251** N0004 NO3

**IL252** C2mim Me-sulfonate

**IL253** C2mim taurinate

**IL254** N1122OH Bu-sulfonate

**IL255** C2mim 4-Me Bn-sulfonate

**IL256** 1-Ph 2,3,5-Me Pyraz Me-sulfonate

**IL257** C03CNim 4-Me Bn-sulfonate

**IL258** N1112OH OHAmEt-solfonate

**IL259** C2mim AAMP-sulfonate

**IL260** 1-Ph 2-Bu 3,5-Me Pyraz Me-sulfonate

**IL261** N1112OH MorOH-sulfonate

**IL262** C2mim BETI 0.724347298 0.135016871 0.011039117 0.016977109

**IL263** C2mim TCN 0.049787492 0.01575507 0.002639789 0.003299832

**IL264** C2mim DCA 0.010928962 0.015577762 0.004079674 0.008013877

**IL265** N1112OH lactate 0.155737705 0.036669697 0 0.003399346

**IL266** C2mim lactate 0.023072253 0.027157974 0 0.00509902

**IL267** S122 TFSI 0.032786885 0.028994252 0.019678426 0.008013877

**IL268** N2OH HCOO 0.281420765 0.0942479 0.007679386 0.007930252

**IL269** Bmim BF4 0.012143291 0.018856181 0.003599712 0.005715476

| **IL270** | C6mim | BF4 | 0.007893139 | 0.012256518 | 0.00887929 | 0.007408704 |
| --- | --- | --- | --- | --- | --- | --- |
| **IL271** | C10mim | BF4 | 0.005464481 | 0.008485281 | 0.072234221 | 0.107858961 |
| **IL272** | Choline | TFSI | 0.149821967 | 0.02422579 | 0.100877193 | 0.026191602 |
| **IL273** | N1116 | TFSI | 0.774370208 | 0.131558183 | 0.057051877 | 0.018190352 |
| **IL274** | N4441 | TFSI | 1.074479737 | 0.090114742 | 0.944009553 | 0.126069646 |
| **IL275** | N4222 | TFSI | 0.709748083 | 0.106773904 | 0.208703728 | 0.103332258 |
| **IL276** | N112Bn | TFSI | 0.867688938 | 0.091975842 | 0.156428287 | 0.107789919 |
| **IL277** | DEME | TFSI | 0.584665936 | 0.032252476 | 0.042987926 | 0.005887841 |
| **IL278** | N2444 | TFSI | 0.770333153 | 0.164423572 | 0.926925643 | 0.051607594 |
| **IL279** | N22214 | TFSI | 0.056587091 | 0.009315344 | 0.010950428 | 0.011089082 |
| **IL280** | N2666 | TFSI | 0.961910753 | 0.044037688 | 0.984086694 | 0.027525805 |
|  |  |  |  |  |  |  |

Sheet 4: Predicted viability

**IL number** cation name anion name predicted viability 1H (av.) predicted viability 24H (av.)

| **IL001** | C2mim | TFSI | 0.030039725 | 0.022208398 |
| --- | --- | --- | --- | --- |
| **IL002** | C4mim | TFSI | 0.700618199 | 0.032493637 |
| **IL003** | C6mim | NTf2 | 0.856637609 | 0.047366647 |
| **IL004** | C8mim | TFSI | 0.727941928 | 0.029786068 |
| **IL005** | C10mim | TFSI | 0.04231212 | 0.024620754 |
| **IL006** | C12mim | TFSI | 0.038440875 | 0.032573238 |
| **IL007** | C4mim | NFSI | 0.666742266 | 0.04973047 |
| **IL008** | C6mim | NFSI | 0.609723097 | 0.061020024 |
| **IL009** | C8mim | NFSI | 0.61668989 | 0.058821666 |
| **IL010** | C4Py | TFSI | 0.530278473 | 0.027594913 |
| **IL011** | N14Pyrr | TFSI | 0.88891697 | 0.050471283 |
| **IL012** | N14Pipe | TFSI | 0.885798437 | 0.110050242 |
| **IL013** | C6Py | TFSI | 0.561772486 | 0.032645104 |
| **IL014** | N13Pipe | TFSI | 0.884938628 | 0.043357752 |
| **IL015** | C4mim | HSO4 | 0.02702879 | 0.014259583 |
| **IL016** | C3mim | BF4 | 0.18034111 | 0.02685814 |
| **IL017** | C2mim | acetate | 0.080970519 | 0.02427921 |
| **IL018** | C4mim | acetate | 0.295296445 | 0.018745415 |
| **IL019** | C2mim | CF3COO | 0.404141341 | 0.024413418 |
| **IL020** | C2mim | C8-sulfate | 0.599920548 | 0.054141357 |
| **IL021** | C4mim | DCA | 0.09071574 | 0.035905574 |
| **IL022** | C4mim | TCN | 0.024621448 | 0.027999233 |
| **IL023** | C6mim | P3C2F5 | 0.633598555 | 0.037411287 |
| **IL024** | C2mim | MePho | 0.342915469 | 0.012342935 |
| **IL025** | C10mim | Br | 0.023853997 | 0.079779245 |
| **IL026** | C8mim | Cl | 0.015906201 | 0.023626776 |
| **IL027** | C8mim | Br | 0.007745881 | 0.037751217 |
| **IL028** | C6mim | Br | 0.014825008 | 0.013951628 |
| **IL029** | P2225 | TFSI | 0.819255521 | 0.821409624 |
| **IL030** | P222(C1O) | NTf2 | 0.803228281 | 0.020420873 |
| **IL031** | P222(C2O) | NTf2 | 0.862521155 | 0.074137506 |

| **IL032** | P2228 | TFSI | 0.99221235 | 0.785683655 |
| --- | --- | --- | --- | --- |
| **IL033** | P4441 | TFSI | 0.840655236 | 0.946726214 |
| **IL034** | P66614 | FSI | 0.486066066 | 0.885738264 |
| **IL035** | P66614 | TFSI | 0.655551106 | 1.083535414 |
| **IL036** | P66614 | CTFSI | 0.633678382 | 0.793060949 |
| **IL037** | P66614 | BETI | 0.660109964 | 1.031099711 |
| **IL038** | P66614 | NFSI | 0.695815148 | 1.082152881 |
| **IL039** | P8888 | NFSI | 0.816219233 | 0.997671734 |
| **IL040** | P8888 | TFSI | 0.75509467 | 0.938708172 |
| **IL041** | P8888 | CTFSI | 0.697362411 | 0.711082431 |
| **IL042** | P8888 | BETI | 0.759852463 | 0.723367973 |
| **IL043** | P66614 | Br | -0.000903954 | 0.001820547 |
| **IL044** | C2mim | BF4 | 0.295369946 | 0.002457765 |
| **IL045** | C4mim | BF4 | 0.168936711 | 0.017477885 |
| **IL046** | C2dmim | BF4 | -0.003676339 | 0.004510107 |
| **IL047** | C2dmim | TFSI | 0.300940486 | 0.013618408 |
| **IL048** | C8mim | BF4 | 0.023405972 | 0.004600571 |
| **IL049** | C2mimOH | BF4 | 0.523850431 | 0.003286546 |
| **IL050** | C2mimOH | TFSI | 0.02331084 | 0.00629356 |
| **IL051** | DEME | BF4 | 0.556585262 | 0.002207598 |
| **IL052** | N14Pyrr | BF4 | 0.294552753 | 0.027490923 |
| **IL053** | C2mim | HSO4 | 0.027424681 | 0.004330401 |
| **IL054** | C0mim | TFSI | 0.179220253 | 0.012979146 |
| **IL055** | dema | TfO | 0.018557504 | 0.001664715 |
| **IL056** | C4mim | I | 0.291387269 | 0.008355773 |
| **IL057** | C4mim | PF6 | 0.509602599 | 0.027188421 |
| **IL058** | C6mim | PF6 | 0.476021333 | 0.026193577 |
| **IL059** | C8mim | PF6 | 0.130471598 | 0.022329536 |
| **IL060** | C4Py | BF4 | 0.094143804 | 0.014928915 |
| **IL061** | C3Py | BF4 | 0.110741431 | 0.009679127 |
| **IL062** | 2-MeC4Py | BF4 | 0.072244212 | 0.021963475 |
| **IL063** | 4-MeC4Py | BF4 | 0.094143804 | 0.019644535 |
| **IL064** | 3-MeC8Py | BF4 | 0.029523158 | 0.016670146 |
| **IL065** | C5mim | BF4 | 0.019133713 | 0.007432512 |

| **IL066** | C4mimCN | BF4 | 0.417932829 | 0.013947134 |
| --- | --- | --- | --- | --- |
| **IL067** | C4dmim | BF4 | 0.033457785 | 0.001681648 |
| **IL068** | C6Py | BF4 | 0.019871385 | 0.016588081 |
| **IL069** | 1-Ph 3,5-Me 2-Pen Pyrazo | BF4 | 0.024227975 | 0.039313951 |
| **IL070** | 1-Ph 3,5-Me 2-Hex Pyrazo | BF4 | 0.033357623 | 0.014586616 |
| **IL071** | C5mim | PF6 | 0.02082329 | 0.012565203 |
| **IL072** | C9mim | PF6 | 0.095626509 | 0.062351312 |
| **IL073** | C2Py | TFSI | 0.056134627 | 0.023536604 |
| **IL074** | N1113 | TFSI | 0.389780593 | 0.023979035 |
| **IL075** | N1114 | TFSI | 0.669005193 | 0.029666617 |
| **IL076** | 2-MeC2Py | TFSI | 0.158627133 | 0.012082742 |
| **IL077** | Ciso4mim | TFSI | 0.312074759 | 0.014831239 |
| **IL078** | C3mim | TFSI | 0.299535447 | 0.030485722 |
| **IL079** | N13Pyrr | TFSI | 0.738152204 | 0.016852951 |
| **IL080** | N2225 | TFSI | 0.881790586 | 0.918223629 |
| **IL081** | 3-MeC3Py | TFSI | 0.506042329 | 0.026847626 |
| **IL082** | 3-MeC4Py | TFSI | 0.333972741 | 0.028341951 |
| **IL083** | 4-MeC4Py | TFSI | 0.372828459 | 0.096186562 |
| **IL084** | 2-MeC3Py | TFSI | 0.275978499 | 0.023087409 |
| **IL085** | NH2-Im-C4-Im-NH2 | TFSI | 0.414580473 | 0.04418073 |
| **IL086** | C10Py | TFSI | 0.054173307 | 0.037238016 |
| **IL087** | N1iso4Pipe | TFSI | 0.891925199 | 0.092989352 |
| **IL088** | 2-Pe3,5-MeC2Py | TFSI | 0.535414962 | 0.031387013 |
| **IL089** | 2-Pe3,5-MeC4Py | TFSI | 0.512277809 | 0.017802726 |
| **IL090** | 2,3,5-MeC6Py | TFSI | 0.557890455 | 0.031387013 |
| **IL091** | 2-Et3,5-MeC6Py | TFSI | 0.523196163 | 0.031387013 |
| **IL092** | 2-Pe3,5-MeC6Py | TFSI | 0.310138964 | 0.029403194 |
| **IL093** | C12Py | TFSI | 0.042402372 | 0.043671507 |
| **IL094** | C14mim | TFSI | 0.072126131 | 0.162706523 |
| **IL095** | N1iso4Pyrr | TFSI | 0.891960002 | 0.035828705 |
| **IL096** | 3-MeCiso4Py | TFSI | 0.660270811 | 0.028568666 |
| **IL097** | C44im | TFSI | 0.616263062 | 0.038141259 |
| **IL098** | 3,5-MeC6Py | TFSI | 0.554151869 | 0.029234399 |
| **IL099** | N2228 | TFSI | 0.873392543 | 0.899005134 |

| **IL100** | C3CN2O2im | TFSI | 0.610913729 | 0.085470949 |
| --- | --- | --- | --- | --- |
| **IL101** | 3-MeC4Py | DCA | 0.111235291 | 0.038493566 |
| **IL102** | P66614 | DCA | 0.315665136 | 0.513618071 |
| **IL103** | 3-MeC2Py | FSI | 0.447352536 | 0.005520941 |
| **IL104** | C6Py | FSI | 0.491071502 | 0.022386868 |
| **IL105** | C4Py | FSI | 0.451960463 | 0.021194901 |
| **IL106** | C2Py | FSI | 0.379637695 | 0.00295858 |
| **IL107** | N13Pyrr | FSI | 0.587303247 | 0.015122011 |
| **IL108** | C2mim | FSI | 0.353183565 | 0.015124138 |
| **IL109** | N14Pyrr | FSI | 0.751467119 | 0.042939214 |
| **IL110** | N14Pyrr | DCA | 0.384915726 | 0.053612317 |
| **IL111** | C6mim | DCA | 0.026467443 | 0.03989359 |
| **IL112** | Choline | NFSI | 0.63019205 | 0.10228325 |
| **IL113** | C2mC1O | DCA | -0.008986502 | 0.007279248 |
| **IL114** | C18dienmim | TFSI | 0.071517372 | 0.080119759 |
| **IL115** | C18monoenmim | TFSI | 0.218012416 | 0.067444706 |
| **IL116** | C4Py | DCA | 0.082865701 | 0.032957808 |
| **IL117** | C3Py | DCA | 0.084564485 | 0.017261215 |
| **IL118** | C6Py | DCA | 0.061585059 | 0.036972868 |
| **IL119** | C3CN2O2im | DCA | 0.040684955 | 0.077221605 |
| **IL120** | C4CNmim | TFSI | 0.255240159 | 0.010924705 |
| **IL121** | N1888 | TFSI | 0.749628395 | 0.738005532 |
| **IL122** | N110 Pyrr | TFSI | 0.89370636 | 0.052302941 |
| **IL123** | N113iso3 | TFSI | 0.817786624 | 0.034461429 |
| **IL124** | N12OH Pyrr | TFSI | 0.594905803 | 0.015021202 |
| **IL125** | N116iso3 | TFSI | 0.927748807 | 0.112015407 |
| **IL126** | N1110iso3 | TFSI | 0.876114947 | 0.104008907 |
| **IL127** | N112OHiso3 | TFSI | 0.725806085 | 0.011527587 |
| **IL128** | N1(OH)2 Pyrr | TFSI | 0.750525215 | 0.029099345 |
| **IL129** | N11114 | TFSI | 0.068031433 | 0.04839208 |
| **IL130** | N22210 | TFSI | 0.839270105 | 0.903566897 |
| **IL131** | N22212 | TFSI | 0.58825299 | 0.878717483 |
| **IL132** | C6Diaza | TFSI | 0.264592029 | 0.02469602 |
| **IL133** | 3,5-MeC4Py | TFSI | 0.560985602 | 0.03041179 |

| **IL134** | 2-Me,5-EtC4Py | TFSI | 0.541757513 | 0.030709214 |
| --- | --- | --- | --- | --- |
| **IL135** | 2,3,5-MeC4Py | TFSI | 0.54632965 | 0.028934893 |
| **IL136** | 3,5-MeC8Py | TFSI | 0.401334289 | 0.024072976 |
| **IL137** | 2,3-MeC8Py | TFSI | 0.550673004 | 0.027552084 |
| **IL138** | 2,3,5-MeC8Py | TFSI | 0.306865036 | 0.018651666 |
| **IL139** | 2,3-MeC4Py | TFSI | 0.498331465 | 0.026884685 |
| **IL140** | 2,4-MeC6Py | TFSI | 0.555952459 | 0.031783277 |
| **IL141** | C4CNdmim | TFSI | 0.345520277 | 0.026346618 |
| **IL142** | C4CNdmim | DCA | 0.007293987 | 0.013929829 |
| **IL143** | C4PyCN | TFSI | 0.332214239 | 0.023085822 |
| **IL144** | C4PyCN | DCA | 0.044892931 | 0.014597718 |
| **IL145** | C4mimCN | DCA | 0.02192883 | 0.003569554 |
| **IL146** | C8mim | TCN | 0.021682215 | 0.017297496 |
| **IL147** | C6mim | TCN | 0.024200882 | 0.03178316 |
| **IL148** | 3,5-MeC8Py | TCN | 0.022635939 | 0.022902721 |
| **IL149** | 2,3-MeC8Py | TCN | 0.022635939 | 0.016576951 |
| **IL150** | P4442 | diEt-Phosphate | 0.732432894 | 0.863518715 |
| **IL151** | C2mim | P3C2F5 | 0.584333525 | 0.030299873 |
| **IL152** | C2mim | diMe-Phosphate | 0.286286526 | 0.010855129 |
| **IL153** | C2mim | diEt-Phosphate | 0.595387811 | 0.017853749 |
| **IL154** | P4441 | Me-sulfate | 0.599820327 | 0.45538578 |
| **IL155** | C2mim | Et-sulfate | 0.158818175 | 0.018067728 |
| **IL156** | C4mim | C8-sulfate | 0.683269765 | 0.114035782 |
| **IL157** | C1mim | Me-sulfate | 0.381970574 | 0.014501973 |
| **IL158** | C4mim | Me-sulfate | 0.339973198 | 0.017824923 |
| **IL159** | L-Ala | C12-sulfate | 0.572167417 | 0.051045367 |
| **IL160** | 2-iso4 Pyrr | C12-sulfate | 0.702996958 | 0.063159072 |
| **IL161** | 1-iso3 3-Me aminium | C12-sulfate | 0.684957082 | 0.053607728 |
| **IL162** | 2-iso3 Pyrr | C12-sulfate | 0.688431693 | 0.052352575 |
| **IL163** | 1-iso4 1-Opr aminium | C12-sulfate | 0.700463966 | 0.058097748 |
| **IL164** | 1,5-iso3 1,5-dioxo5 aminium | C12-sulfate | 0.689899537 | 0.063108812 |
| **IL165** | 2-EtC1Py | Me-sulfate | 0.288920873 | -0.004364559 |
| **IL166** | 2-EtC2Py | Et-sulfate | 0.283866313 | 0.010139924 |
| **IL167** | C1Py | Me-sulfate | 0.367951585 | 0.009646101 |

| **IL168** | 3-MeC1Py | Me-sulfate | 0.368437213 | 0.009646101 |
| --- | --- | --- | --- | --- |
| **IL169** | C2Py | Et-sulfate | 0.175313508 | 0.013418866 |
| **IL170** | C2mim | Me-sulfate | 0.271111327 | 0.004330401 |
| **IL171** | Cbnmim | Me-sulfate | 0.171164837 | 0.007311445 |
| **IL172** | 3-MeC2Py | Et-sulfate | 0.283866313 | 0.012875455 |
| **IL173** | N14Pyrr | Me-sulfate | 0.602606256 | 0.032315041 |
| **IL174** | N2221 | Me-sulfate | 0.442966862 | 0.007115098 |
| **IL175** | N12Pyrr | Et-sulfate | 0.482428995 | 0.009876891 |
| **IL176** | C4pro-CNim | C12-sulfate | 0.592906423 | 0.076813616 |
| **IL177** | C10pro-CNim | C12-sulfate | 0.641724052 | 0.080933574 |
| **IL178** | C8pro-CNim | C10-sulfate | 0.590310433 | 0.080546311 |
| **IL179** | C6pro-CNim | C12-sulfate | 0.590310433 | 0.080546311 |
| **IL180** | C0mim | HSO4 | 0.457394273 | 0.008464531 |
| **IL181** | C0Buim | HSO4 | 0.352764882 | 0.012227527 |
| **IL182** | C2mim | MeOEtOEt-sulfate | 0.592828683 | 0.02852193 |
| **IL183** | N24Pyrr | Et-sulfate | 0.637023559 | 0.800539886 |
| **IL184** | C3mim | Me-sulfate | 0.303423155 | 0.013531347 |
| **IL185** | C6mim | Et-sulfate | 0.318915315 | 0.000906586 |
| **IL186** | C8mim | MeOEtOEt-sulfate | 0.601104561 | 0.048480676 |
| **IL187** | N1(EtOH)3 | Me-sulfate | 0.613470799 | 0.054441659 |
| **IL188** | C2etim | Et-sulfate | 0.084823584 | 0.017411266 |
| **IL189** | N0002 | HSO4 | 0.408078646 | 0.012539498 |
| **IL190** | C4mim | MeOEtOEt-sulfate | 0.656581575 | 0.037822094 |
| **IL191** | N1122OH | Et-sulfate | 0.587774202 | 0.009237079 |
| **IL192** | N1124 | Et-sulfate | 0.621406253 | 0.03663888 |
| **IL193** | N0022 | HSO4 | 0.369839714 | 0.014937453 |
| **IL194** | N0111 | HSO4 | 0.283899704 | 0.010260284 |
| **IL195** | N0222 | HSO4 | 0.040073763 | 0.005284839 |
| **IL196** | C2Py | TfO | 0.394579676 | 0.013843033 |
| **IL197** | C4Py | TfO | 0.405131173 | 0.018404455 |
| **IL198** | C2mim | TfO | 0.384601984 | 0.020879178 |
| **IL199** | C4mim | TfO | 0.456621375 | 0.023975813 |
| **IL200** | N14Pyrr | TfO | 0.607580831 | 0.036445645 |
| **IL201** | 3-MeC4Py | TfO | 0.140616759 | 0.018433651 |

| **IL202** | C42im | TfO | 0.068112254 | 1.04707E-05 |
| --- | --- | --- | --- | --- |
| **IL203** | 2,3-MeC4Py | TfO | 0.401069344 | 0.010814413 |
| **IL204** | C6mim | TfO | 0.135776319 | 0.023010308 |
| **IL205** | C43CNim | TfO | 0.453303627 | 0.015916368 |
| **IL206** | C63CNim | TfO | 0.300653429 | 0.006167011 |
| **IL207** | C83CNim | TfO | 0.06767859 | 0.013028656 |
| **IL208** | 2,3-MeC8Py | TfO | 0.085047816 | 0.016903601 |
| **IL209** | P66614 | TfO | 0.501308104 | 0.63221843 |
| **IL210** | C4mim | Br | 0.368002047 | 0.019476638 |
| **IL211** | C43CNim | Br | 0.387613445 | 0.018012818 |
| **IL212** | C63CNim | Br | 0.012444083 | 0.015002943 |
| **IL213** | C83CNim | Br | 0.012444083 | 0.015180624 |
| **IL214** | P8888 | Br | 0.175045557 | 0.363369263 |
| **IL215** | C3all3CN | Cl | 0.013833571 | 0.010196421 |
| **IL216** | C2OH3CN | Cl | 0.013833571 | 0.016392352 |
| **IL217** | C6mim | Cl | 0.027824902 | 0.018559054 |
| **IL218** | C6OH0im | Cl | 0.025489582 | 0.00603808 |
| **IL219** | CBnmim | Cl | 0.018325824 | 0.013895305 |
| **IL220** | C43CNim | Cl | 0.014491358 | 0.011562331 |
| **IL221** | C6OHmim | Cl | 0.018992849 | 0.034759103 |
| **IL222** | C63CNim | Cl | 0.006870805 | 0.022650181 |
| **IL223** | C10mim | Cl | 0.053921904 | 0.063721659 |
| **IL224** | C46OHim | Cl | 0.044231411 | -0.007942982 |
| **IL225** | C83CNim | Cl | 0.040026544 | 0.018252206 |
| **IL226** | P4448 | Cl | 0.03415987 | 0.031306986 |
| **IL227** | P66614 | Cl | 0.078796195 | 0.028095593 |
| **IL228** | N0002 | acetate | 0.286459389 | 0.033334328 |
| **IL229** | N0111 | acetate | 0.24511573 | 0.031101888 |
| **IL230** | N0003 | acetate | 0.324514317 | 0.01508108 |
| **IL231** | N0002 | glycolate | 0.20690672 | 0.007527316 |
| **IL232** | N0004 | acetate | 0.370205355 | -0.003348165 |
| **IL233** | N0022 | acetate | 0.247616793 | -0.009661098 |
| **IL234** | N0112 | acetate | 0.191955788 | 0.029070921 |
| **IL235** | N0012OH | acetate | 0.281206061 | 0.008786896 |

| **IL236** | C0mim | acetate | 0.310022781 | 0.03662543 |
| --- | --- | --- | --- | --- |
| **IL237** | N0222 | acetate | 0.147229888 | 0.034146735 |
| **IL238** | N1112OH | glycinate | 0.124174389 | 0.003498015 |
| **IL239** | C03CNim | acetate | 0.17912845 | 0.009325377 |
| **IL240** | C3mim | acetate | 0.125175007 | 0.023212443 |
| **IL241** | C2mim | ammonioacetate | 0.055000437 | 0.006131157 |
| **IL242** | N14Pyrr | acetate | 0.54782402 | 0.05541387 |
| **IL243** | C5mim | acetate | 0.192822371 | 0.012632789 |
| **IL244** | C6mim | acetate | 0.179077504 | 0.025026251 |
| **IL245** | CEtOEt3CN | CF3COO | 0.472868821 | 0.029773486 |
| **IL246** | C4mim | ibuprofenate | 0.690802876 | 0.053475208 |
| **IL247** | P66614 | acetate | 0.490195394 | 0.519889131 |
| **IL248** | N0002 | NO3 | 0.269439317 | 0.016598959 |
| **IL249** | N0002OH | NO3 | 0.283400184 | 0.007399044 |
| **IL250** | Pyrr | NO3 | 0.235787607 | 0.012964415 |
| **IL251** | N0004 | NO3 | 0.338920624 | 0.011086785 |
| **IL252** | C2mim | Me-sulfonate | 0.048463694 | 0.01258656 |
| **IL253** | C2mim | taurinate | 0.30646519 | 0.004128575 |
| **IL254** | N1122OH | Bu-sulfonate | 0.674765775 | 0.025321917 |
| **IL255** | C2mim | 4-Me Bn-sulfonate | 0.582642671 | 0.023969911 |
| **IL256** | 1-Ph 2,3,5-Me Pyraz | Me-sulfonate | 0.058704254 | 0.019689984 |
| **IL257** | C03CNim | 4-Me Bn-sulfonate | 0.494937445 | 0.024388208 |
| **IL258** | N1112OH | OHAmEt-solfonate | 0.488507377 | 0.040978482 |
| **IL259** | C2mim | AAMP-sulfonate | 0.601779379 | 0.04030112 |
| **IL260** | 1-Ph 2-Bu 3,5-Me Pyraz | Me-sulfonate | 0.062387357 | 0.010916014 |
| **IL261** | N1112OH | MorOH-sulfonate | 0.488507377 | 0.05107386 |
| **IL262** | C2mim | BETI | 0.712637424 | 0.01116692 |
| **IL263** | C2mim | TCN | 0.046645885 | 0.003654827 |
| **IL264** | C2mim | DCA | 0.023997577 | 0.004330401 |
| **IL265** | N1112OH | lactate | 0.147693325 | 0.000389939 |
| **IL266** | C2mim | lactate | 0.052562131 | 0.003559355 |
| **IL267** | S122 | TFSI | 0.037700268 | 0.019390272 |
| **IL268** | N2OH | HCOO | 0.273726236 | 0.005862967 |
| **IL269** | Bmim | BF4 | 0.019133713 | 0.006352852 |

**IL270** C6mim BF4 0.022314864 0.01177497

**IL271** C10mim BF4 0.006771363 0.066977956

**IL272** Choline TFSI

**IL273** N1116 TFSI

**IL274** N4441 TFSI

**IL275** N4222 TFSI

**IL276** N112Bn TFSI

**IL277** DEME TFSI

**IL278** N2444 TFSI

**IL279** N22214 TFSI

**IL280** N2666 TFSI
